# Adverse Outcome Pathways Applied to Space Radiation Research

**DOI:** 10.1002/em.70031

**Published:** 2025-09-08

**Authors:** Vinita Chauhan, Veronica S. Grybas, Devyn Hoopfer, Casey Higginson, Elizabeth A. Ainsbury, Omid Azimzadeh, Afshin Beheshti, Steve Blattnig, Marjan Boerma, Sylvain V. Costes, Stephen Doty, Christelle Adam‐Guillermin, Nobuyuki Hamada, Patricia Hinton, Janice L. Huff, Robert Reynolds, Ruth C. Wilkins, Scott Wood, Carole L. Yauk

**Affiliations:** ^1^ Consumer and Clinical Radiation Protection Bureau, Health Canada Ottawa Canada; ^2^ Radiation Effects Department, Radiation, Chemical and Environmental Hazards Directorate UK Health Security Agency Birmingham UK; ^3^ Environmental Research Group Within the School of Public Health Faculty of Medicine at Imperial College of Science, Technology and Medicine London UK; ^4^ Federal Office for Radiation Protection (BfS) Section Radiation Biology Neuherberg Germany; ^5^ McGowan Institute for Regenerative Medicine ‐ Center for Space Biomedicine, Department of Surgery University of Pittsburgh Pittsburgh Pennsylvania USA; ^6^ Stanley Center for Psychiatric Research Broad Institute of MIT and Harvard Cambridge Massachusetts USA; ^7^ NASA Langley Research Center Hampton Virginia USA; ^8^ University of Arkansas for Medical Sciences Little Rock Arkansas USA; ^9^ NASA Ames Research Center Space Biosciences Research Branch Mountain View California USA; ^10^ Hospital for Special Surgery Research Institute New York New York City USA; ^11^ PSE‐SANTE/SDOS/LMDN, Institut de Radioprotection et de Sureté Nucléaire (IRSN) Provence France; ^12^ Biology and Environmental Chemistry Division, Sustainable System Research Laboratory Central Research Institute of Electric Power Industry (CRIEPI) Chiba Japan; ^13^ Canadian Forces Environmental Medicine Establishment Toronto Ontario Canada; ^14^ KBR, NASA Johnson Space Center Houston Texas USA; ^15^ NASA Johnson Space Center Houston Texas USA; ^16^ Department of Biology University of Ottawa Ottawa Ontario Canada

**Keywords:** adverse outcome pathway, AOP, OECD, radiation, radiation protection

## Abstract

Long‐duration spaceflight exposes astronauts to various stressors that can alter human physiology, potentially causing immediate and long‐term health effects. These stressors can damage biomolecules, cells, tissues, and organs, leading to adverse outcomes. Developing adverse outcome pathways (AOPs) relevant to radiation exposure can guide research priorities and inform risk assessments of future space exploration activities. Through expert consultation, we developed an AOP network linking 18 key events (KEs) to four non‐cancer outcomes: learning and memory impairment, bone loss, abnormal vascular remodeling, and cataract development. A novel scoping review methodology informed the evidence evaluation and supported causal linkages between two KEs. The AOP network begins with the molecular initiating event (MIE) of energy deposition onto cells, which may trigger oxidative stress and DNA damage. If DNA damage is misrepaired, it could lead to gene mutations or chromosomal aberrations. In cases where these occur in critical cell cycle genes, there is a possibility of uncontrolled cellular proliferation. Persistent KEs may contribute to the activation of tissue‐resident cells, suppression of anti‐inflammatory processes, and promotion of chronic inflammation. This inflammatory cycle, potentially driven by mitochondrial dysfunction and immune cell activation, could lead to cell death and tissue damage. Over time, this accumulation of damage might contribute to organ‐specific adverse outcomes associated with radiation exposure. This AOP network consolidates knowledge across biological levels and identifies gaps in understanding causal relationships. It aims to guide research for space traveler risk models and can also apply to other radiation exposure scenarios, such as in medical or occupational settings.

## 1 Introduction

Space presents a complex environment for astronauts, subjecting them to multiple stressors, including radiation, microgravity or hypergravity, isolation, confinement, and altered CO_2_ levels. Evaluating the risks associated with these stressors is inherently challenging. Space agencies like NASA use integrated design tools and risk models to establish safety regulations designed to minimize these risks. Among the many stressors, radiation is of particular concern. For radiation, baseline risks are assessed based on population incidence and mortality data, which are then applied to calculate the excess risk posed by space radiation. This excess risk is adjusted using specific weighting factors (Huff et al. 2022). However, a key challenge in these models is the variety of radiation types astronauts may encounter, including energetic photons, electrons, protons, helium nuclei, and high‐charge, high‐energy (HZE) ions, all of which can cause biological damage at doses equal to or even lower than those found at Earth's surface (Huff et al. 2022; Boerma et al. 2016).

In addition to radiation, microgravity affects biological systems, influencing blood flow, the central nervous system (CNS), circadian rhythms, and bone density (Man et al. 2022; Afshinnekoo et al. 2020). Understanding these effects in complex, mixed‐exposure scenarios is particularly difficult, especially given that most astronauts have not traveled beyond Earth's magnetosphere, resulting in a lack of deep space health data (Huff et al. 2022). Compounding this issue is the lack of chronic exposure studies involving high‐linear energy transfer (LET) radiation in humans, requiring extrapolation from in vivo and in vitro models, which limits the strength of predictive results (Antonsen et al. 2023).

To address some of these challenges, a framework is needed to integrate mechanistic knowledge on the health outcomes resulting from the various hazards encountered in space. The Organisation for Economic Co‐operation and Development (OECD) Adverse Outcome Pathway (AOP) framework offers a structured approach for organizing scientific data into sequences of causally related events that lead to adverse health outcomes (Ankley et al. 2010; OECD 2018). An AOP begins with a molecular initiating event (MIE), followed by intermediate key events (KEs), and culminates in an adverse outcome (AO). These KEs are linked by key event relationships (KERs), with causality assessed using the modified Bradford‐Hill criteria, which include biological plausibility, essentiality, and empirical evidence such as dose‐, incidence‐and time‐concordance (Becker et al. 2015). The strength of these relationships is evaluated through a weight of evidence analysis for each KER (Villeneuve et al. 2014). Although AOPs are typically simplified to focus on measurable KEs, their linear structure allows for the development of networks where multiple MIEs can converge to produce the same AO. This flexibility makes the AOP framework particularly effective for assessing the complex, multi‐stressor environment of space (Table 1).

**TABLE 1:** Addressing space travel challenges with adverse outcome pathways (AOPs).

Challenge	How AOPs address the challenge
Complex multi‐stressor environment	Space‐relevant stressors (e.g., radiation, altered gravity, altered CO_2_ levels) can be mapped to events in AOPs to analyze how they interact and impact the adverse outcomes (AOs)
Sparse quantitative data	By organizing data from animal and in vitro models around mechanistic endpoints, AOPs enhance extrapolation to human health risks and improve predictive accuracy. Data organized through AOPs can provide quantitative information to inform risk models for multiple AOs and aid in translating findings to acceptable dose estimates
Complex non‐cancer outcomes	AOPs simplify the broad spectrum of pathophysiologies for non‐cancer outcomes (e.g., cardiovascular disease, neurological disorders) by focusing on data‐rich mechanistic endpoints, facilitating targeted research and understanding
Relevant experiments	AOPs provide a framework for experimental design around key mechanistic endpoints that are assessed through various initiating events to understand their impact on the severity and progression of biological effects

While AOPs have traditionally been used to describe chemical and ecological outcomes, there is growing interest in applying this framework to radiation research, particularly in the context of space (Chauhan et al. 2024; NCRP 2020; Chauhan et al. 2019). For example, the AOP for lung cancer caused by radon exposure has been endorsed by the Nuclear Energy Agency (NEA) and other OECD bodies (https://aopwiki.org/aops/272). Since 2021, the NEA's Radiation/Chemical AOP Joint Topical Group has worked to integrate AOPs into radiation research and risk assessments (Chauhan, Beaton, et al. 2022; Chauhan, Hamada, et al. 2022).

Building on these efforts, an AOP network was developed for non‐cancer health outcomes relevant to space travel (Kozbenko et al. 2024; Carrothers et al. 2024; Sleiman et al. 2024; Sandhu et al. 2024). The AOs selected for this network include bone loss (https://www.aopwiki.org/aops/482), cataracts (https://www.aopwiki.org/aops/478), learning and memory impairment (https://www.aopwiki.org/aops/483), and abnormal vascular remodeling (https://www.aopwiki.org/aops/470). It is important to note that this is not an exhaustive list, and future AOP development should also address other AOs, such as reproductive effects, musculoskeletal atrophy, spaceflight‐associated neuro‐ocular syndrome (SANS) (Mehare et al. 2024), and cancer (Turker et al. 2017; Sasi et al. 2015; Patel et al. 2020; Kennedy et al. 2018). While cancer falls outside the scope of the current network, it is covered by other AOPs (e.g., AOP #272 (Chauhan et al. 2021; Sherman et al. 2023); AOP #432 (Klokov et al. 2022)) and could eventually be integrated into this network. The following sections outline the development process of this AOP network, summarizing key publications, highlighting knowledge gaps, and providing actionable recommendations for future research.

## 2 Strategy for Evidence Collection for AOP Development

**FIGURE 1:**
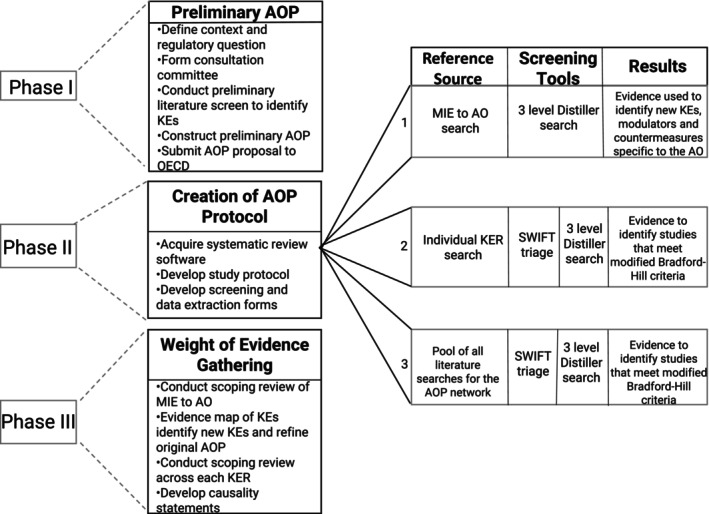
Workflow for the phases of the project and evidence gathering to support key event relationships using the Bradford‐Hill criteria.

**FIGURE 2:**
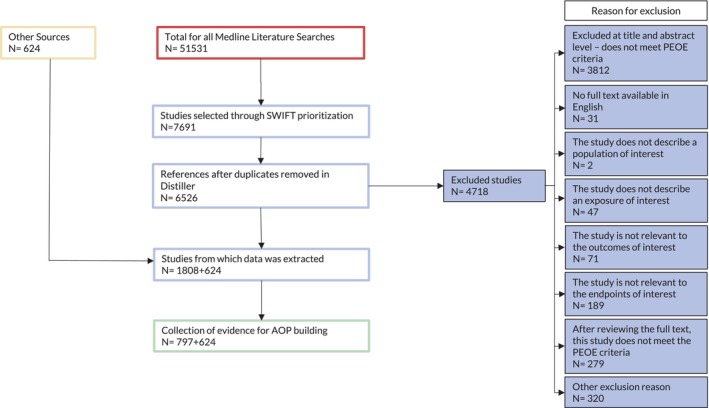
PRISMA diagram depicting reference screening of all AOs during the scoping review as described in Kozbenko et al. (2022). “Other sources” refers to additional papers supporting the AOP outside of the Medline literature searches (e.g., from subject matter experts and reference sections of review articles). References could be excluded for multiple exclusion reasons simultaneously. Literature searches for each AO may have identified the same studies.

The strategy used for building the AOP network has been described (Kozbenko et al. 2022). Traditionally, AOPs are built using a narrative approach. However, in this initiative, elements of systematic review were incorporated and a transparent approach to data retrieval and evidence evaluation was undertaken. Briefly, the AOP workflow was divided into three phases (Figure 1). Of these, Phase I (Preliminary AOP development) involved the construction of a hypothetical AOP through a subject matter consultation and a manual literature search of ~100 studies relevant to space travel using search engines (e.g., Google Scholar and PubMed) and databases and reports provided by NASA and CSA. This initial search identified the four AOs along with 40 adjacent KERs (two consecutive KEs in an AOP) and 18 non‐adjacent KERs (not sequential), which were justified based on the retrieval of limited empirical evidence in the form of dose‐, time‐, and incidence‐concordance. In Phase II (Creation of evidence retrieval protocols), a librarian performed structured literature searches across each KER. A study protocol was developed and registered (osf.io/t9amw) using established guidelines from the EVNINT PRISMA‐SM‐P report (Elsevier 2017). In Phase III (Weight of evidence gathering and assessment), the study protocol was tested, and amendments were made to Phase II accordingly, which resulted in three workflows describing increasingly efficient processes for AOP evidence collection (Kozbenko et al. 2022) (Figure 1). Databases generated by the librarian were screened in SWIFT Review (Sciome Workbench for Interactive computer‐Facilitated Text‐mining) www.sciome.com/swift‐review/ released 08.28.2019: version 1.43, which was used to prioritize studies based on a population, exposure, and outcome/endpoint (PEO/E) statement, and the relevant data files were imported into Distiller (DistillerSR, Ontario, Canada), an artificial intelligence (AI) based software. This method reduced the number of full‐text article reviews and focused screeners' attention on prioritized search results. A PRISMA diagram depicting reference screening for all AOs is found in Figure 2. Approaching AOP development in a systematic manner and using AI along with multi‐level screening allowed for less bias, increased transparency, and reduced burden on human screeners (Kozbenko et al. 2022).

## 3 Building the AOP Network

**FIGURE 3:**
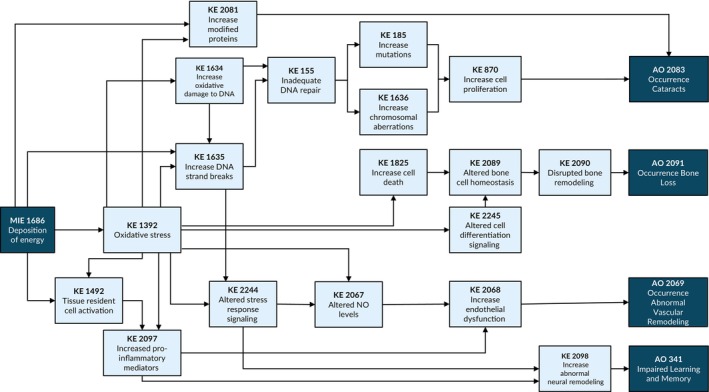
AOP network of four intersecting adverse outcomes linked by multiple adjacent key events beginning with deposition of energy.

The three‐tiered screening led to the development of an initial AOP network (Figure 3). This hypothetical AOP network illustrates the sequential chain of causally linked events from an MIE to measurable KEs across levels of biological organization to non‐cancer outcomes. The KEs were refined as the complete screening of the literature was undertaken. Though the principles of AOP development indicate that an AOP should be stressor‐agnostic, radiation studies were prioritized, as it is among the most detrimental hazards encountered in space and of high interest in other radiation protection sectors (e.g., environmental, occupational and medical exposures). Future work could involve a complete assessment of all stressors and other AOs relevant to the space environment.

Ten out of 18 KEs (KE #155, KE #185, KE #870, KE #1392, KE #1492, KE #1493, KE #1634, KE #1635, KE #1636, KE #1686) in the AOP network were reused from existing endorsed AOPs in the AOP‐Wiki (www.aopwiki.org). This includes the AO of learning and memory impairment (KE #341). The AOP network was supported by a wide range of radiation stressors and is therefore not specific to any particular exposure parameter (i.e., dose‐range, dose rate, or radiation quality). This aligns with AOP principles, as AOPs are informed by the understanding of the upstream biological perturbations in the context of the downstream KEs, and these perturbations can be induced by many stressors. Some KEs are broad in scope (capture multiple biological effects) as the complexity of some molecular and cellular changes can be difficult to delineate, and parallel mechanisms may be operating in concert to cause the downstream effects. As more knowledge emerges, these KEs can be split into more focused KEs if required for specific radiation exposures. Given the often limited data available for adjacent KERs, the incorporation of non‐adjacent KERs in AOPs becomes essential to address gaps. Many KEs were identified that relate to both radiation exposures and microgravity. Where appropriate, studies from both stressors were captured within the AOP evidence evaluation, particularly for KEs that are not directly linked to the MIE. Further integration of this network (e.g., to include MIEs related to other space stressors) with other relevant AOPs can advance the quantitative understanding of the impacts of space travel.

## 4 Defining Components of the AOP

### 4.1 MIEs

In building the AOP, considerable discussions were directed to the selection of the initiating events for the project. Space is a complex environment where altered gravity and radiation are important contributors to astronaut health. An MIE for microgravity was discussed but it was difficult to define. Future iterations of the AOP could improve this aspect. In terms of radiation, the most relevant particles to space travel are protons, neutrons, alpha particles, and heavy ions, either ejected during solar particle events (SPEs) or as components of galactic cosmic rays (GCR) (Townsend 2005; Chancellor et al. 2014). Another major concern for crew exposure is solar cosmic radiation, specifically the unpredictable occurrence of large SPEs. During a Mars mission, crew members will likely be exposed to several SPEs, and spacecraft shielding will not be able to completely protect from this exposure (Chancellor et al. 2014). Unlike most SPE particles that can be stopped by adequate shielding, GCR particles penetrate the spacecraft and other surfaces, producing secondary particles including neutrons, lighter nuclei, electrons and gamma rays (Warden and Bayazitoglu 2019). Therefore, to encompass the various energies and dose rates that astronauts may be exposed to, the MIE was defined as “deposition of energy”, which is relevant to all types of radiation. With this MIE, the AOP network will have broad applicability to other domains outside of the space field (i.e., medical, occupational, environmental). Although the AOP begins with deposition of energy, other MIEs can be included in the future to expand the network, as AOPs are living documents that evolve as the science advances.

### 4.2 AOs

Non‐cancer outcomes were the initial focus of the AOP network because while cancer risks from various stressors, including radiation, are extensively studied and relatively well understood, non‐cancer endpoints often remain less explored. The four AOs that were interconnected in the network included bone loss (https://www.aopwiki.org/aops/482), cataracts (https://www.aopwiki.org/aops/478), learning and memory impairment (https://www.aopwiki.org/aops/483), and occurrence of abnormal vascular remodeling (https://www.aopwiki.org/aops/470). As described below, there is a sufficient body of human studies to support their regulatory significance.

Bone loss is a well‐documented AO related to space flight due to changes in microgravity (Willey et al. 2011). However, growing evidence suggests that bone loss can also result from radiation exposure (Wright 2018; Willey et al. 2013). The available human data for bone loss come primarily from studies on returning astronauts (Stavnichuk et al. 2020). Examining astronauts from Apollo, International Space Station (ISS), and various other low Earth orbit missions revealed bone loss in the lower skeleton and preserved bone in the upper skeleton (Stavnichuk et al. 2020). Clinical studies on radiotherapy patients with pelvic, breast, and rectal cancers using high doses of fractionated ionizing radiation showed an increased risk of fractures to hips, ribs, femoral head, and neck arising 5–10 years post‐radiotherapy (Baxter et al. 2005; Willey et al. 2011; Williams and Davies 2006; Oeffinger et al. 2006). It is established that chronic low‐dose radiation contributes to bone loss, but there is less research on the effects of long‐duration ionizing radiation exposure (Stavnichuk et al. 2020).

Radiation exposures are also associated with abnormal vascular remodeling and an increased risk of CVD (Boerma et al. 2016; Little et al. 2023). High‐dose clinical trials, including studies with radiotherapy patients (Zou et al. 2019), indicate an increased CVD risk. Low‐dose retrospective and prospective cohort studies utilize Japanese atomic bomb survivors, protracted occupational exposures, and Chornobyl survivors. Findings from such epidemiological studies include an increased risk of CVD morbidity (Zielinski et al. 2009) and mortality (Gillies et al. 2017; Ivanov et al. 2006; Kashcheev et al. 2017) but study designs have historically limited the strength of these studies (e.g., small sample size). Atomic bomb survivor studies have identified the dose influencing CVD outcome risk (Ozasa et al. 2012; Preston et al. 2003; Shimizu et al. 2010; Takahashi et al. 2017). A recent meta‐analysis of 93 epidemiological studies highlights significant uncertainties regarding confounding factors that may affect the association between radiation and CVD (Little et al. 2023). Although excess radiation risk has not been reported in astronauts, it is a priority for investigation (Patel et al. 2020).

Evidence suggests that radiation exposure can lead to reduced cognitive function (Collett et al. 2020). However, the exact mechanisms underlying this remain poorly understood (Pasqual et al. 2021). Cognitive impairment related to radiation exposure has been observed in various populations, including radiotherapy patients (Krull et al. 2018), atomic bomb survivors (Yamada et al. 2016), and occupationally exposed individuals such as nuclear workers, uranium miners, medical workers, Mayak workers, and Chornobyl clean‐up workers (Azizova et al. 2020; Lestaevel et al. 2016; Taormina et al. 2008). Additionally, animal studies have contributed to this understanding (Cekanaviciute et al. 2018) but the direct mechanisms have yet to be fully elucidated. The determination of cognitive effects is complicated by various assessment methods used and uncertainty around the effects of low to moderate doses (Pasqual et al. 2021).

Ionizing radiation is also a well‐established cataractogen (Ainsbury et al. 2016), supported by abundant evidence from animal and human epidemiological studies. Cohorts of atomic bomb survivors (Minamoto et al. 2004), Mayak workers (Azizova et al. 2016), Chornobyl clean‐up workers (Worgul et al. 2007), astronauts (Chylack Jr et al. 2009, 2012), and aviators (Jones et al. 2006) were used to derive evidence for the International Commission on Radiological Protection (ICRP) to set eye lens dose limits (ICRP 2012; Rao 2016). Posterior subcapsular cataracts are the most common type of cataract that develops after exposure to ionizing radiation. While cataracts develop after either acute or chronic exposure to ionizing radiation (Hamada, Azizova, and Little 2020), there are relatively limited data from astronauts on the role of cosmic radiation in the increased risk of cataracts. Further research is needed at lower doses and dose rates to fully understand the mechanisms and develop effective countermeasures.

## 5 Description of the KEs

### 5.1 Macromolecular/Cellular Events

**FIGURE 4:**
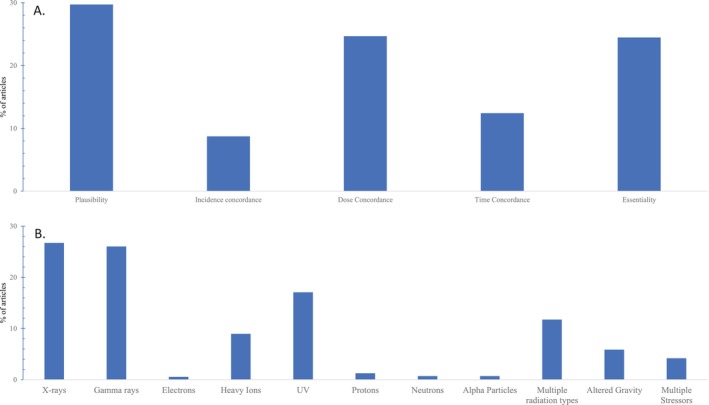
Summary of the (A) Evidence streams, and (B) Stressor types used to support the AOP.

The initial screening of the literature identified biological events leading to the four AOs of interest. Not all are included in the final AOP network due to either the lack of a well‐defined measurement method or the lack of empirical evidence to support connectivity to another KE. Existing KEs used in our proposed pathways are linked to AOP #15, 72, 136, 139, 141, 216, 238, 272, 293, 294, 296, 303, 322, 397, 409, 420, 432, 443, and 451 in the AOP‐Wiki. The KEs with strong biological plausibility are described below, with further details found in the individual AOP reports (Kozbenko et al. 2024; Carrothers et al. 2024; Sleiman et al. 2024; Sandhu et al. 2024). Empirical evidence to support each KER is drawn predominantly from radiation stressors (accounting for ~76% of the studies), altered gravity (~17%), and multiple space stressors (~8%) (Figure 4). It is important to note that not all stressors reported support each KER; for details on individual KERs, refer to the AOP‐Wiki.

The AOP network begins with deposition of energy, which refers to events where energetic subatomic particles, nuclei, or electromagnetic radiation deposit energy in the media through which they transverse. The energy may either be sufficient (e.g., ionizing radiation) or insufficient (e.g., non‐ionizing radiation) to ionize atoms or molecules. Energy deposition into matter may lead to a biological cascade of damage depending on the type (photons, electrons, protons, neutrons, etc.) and energy of the radiation. Densely ionizing radiation produces more irreparable clustered damage (Mavragani et al. 2019). Energy absorption may occur within 10^−17^ to 10^−13^ s following radiation exposure to living cells (Bouten et al. 2021). However, not all deposited energy initiates a path to disease/adversity as sufficient damage is required to overwhelm the natural homeostatic processes for defense.

Deposited energy may directly or indirectly damage macromolecules within the cell. The indirect damage is initiated with the breakage of water molecules to hydrogen and hydroxyl ions. This hydrolysis generates free radicals or reactive oxygen species (ROS) which may occur within 10^−10^ to 10^−6^ s (Bouten et al. 2021). The further interaction of free radicals with nitric oxide (NO) molecules within cells, produced from nitric oxide synthase (NOS), reduces NO levels and leads to reactive nitrogen species (RNS) generation. Together, the accumulation of free radical damage through various sources (e.g., radiolysis of water, mitochondrial damage) (Desouky et al. 2015; Mittal et al. 2014; Soloviev and Kizub 2019) may overwhelm antioxidant enzymes and free radical scavengers. Low antioxidant production/level then leads to a state of oxidative stress (AOP #470) (Slezak et al. 2017; Tahimic and Globus 2017; Wang et al. 2019).

Oxidative stress may damage macromolecules (DNA, lipids, proteins) leading to by‐products (e.g., through peroxidation) that may activate resident cells, causing an increase in pro‐inflammatory mediators (Chang et al. 2017; Mavragani et al. 2019; Hladik and Tapio 2016; Ping et al. 2020). In turn, cells may transmit damage signals through gap junction‐mediated intercellular channels and by secreting cytokines and ROS into the extracellular matrix, leading to the further spread of oxidative stress, damage, and altered signaling in neighboring non‐irradiated cells (Little et al. 2023; Hamada et al. 2007, 2011). In the minutes to hours following the initial damage, ROS may activate nuclear factor κB (NF‐κB), leading to the production of various pro‐inflammatory mediators, including tumor necrosis factor α (TNF‐α), interleukin 1 (IL‐1), and interleukin 6 (IL‐6). Exposure to ionizing radiation may also release alarmins and damage associated molecular patterns (DAMPs) that promote activation of nearby resident immune cells (AOP #483) such as macrophages and dendritic cells in the brain. Immune cell activation through pattern recognition of the DAMPs may promote inflammation (AOP #483), cause regeneration of the affected cells and tissues, and promote proliferation and recruitment of additional immune cells (Chen et al. 2016; Collett et al. 2020; Cucinotta et al. 2014; Hladik and Tapio 2016; Schaue et al. 2015). This activation may alter the activity of receptors and disrupt normal cellular signaling processes. Altered signaling is a shared KE across several AOPs and is a central connector in the network.

Cell signaling, particularly the stress response, may vary depending on cell type and anatomical location. In response to DNA damage, many signaling pathways are initiated. Resultant changes in signaling pathways explored in our AOP network include: the phosphatidyl inositol 3‐kinase (PI3K)/Protein kinase B (Akt) pathway, cAMP response element‐binding protein (CREB) pathway, the p53/p21 pathway, as well as the mitogen‐activated protein kinase (MAPK) family pathways, including c‐Jun N‐terminal kinase (JNK), extracellular signal‐regulated kinase (ERK) and p38 (Lehtinen and Bonni 2006; Ramalingam and Kim 2012). Together, these alterations may result in changes in cell cycle, senescence, apoptosis, and proliferation (Betlazar et al. 2016). For instance, in many cell types, phosphorylation of p53 may occur, stabilizing the protein and propagating cellular senescence or triggering a caspase cascade that results in apoptosis (Hein et al. 2014). In endothelial cells, the PI3K/Akt/mammalian target of rapamycin (mTOR) signaling pathway may modulate the levels of endothelial nitric oxide synthase in response to different radiation exposure scenarios (Yentrapalli et al. 2013; Azimzadeh et al. 2015, 2017, 2021; Vieira Dias et al. 2018; Hamada, Kawano, et al. 2020). Similarly, in bone cells, the activity of osteoblasts and osteoclasts, governed by signaling pathways related to cell differentiation, dictates bone generation and resorption. Specifically, the binding of receptor activator of NF‐κB (RANK) to its ligand (RANK‐L) triggers the differentiation of hematopoietic stem cells into pre‐osteoclasts and osteoclasts (Donaubauer et al. 2020; Smith 2020). Conversely, osteoprotegerin (OPG) may competitively bind to RANK‐L. In turn, RANK‐L inhibits osteoclast differentiation and reduces bone resorption, leading to bone loss. Thus, the balance between cell death and proliferation across various tissues may be significantly influenced by direct damage to proteins, DNA, or cells and is regulated by many concerted signaling pathways.

Alongside the indirect damage caused by energy deposition, direct damage may also occur. Deposition of energy leading to ionization events may also concurrently cause direct bond breakage of proteins and DNA molecules, leading to various molecular modifications (Fochler and Durchschlag 1997). A multitude of reviews suggests that at sufficient levels of ionizing radiation, increased protein modifications (AOP #478) may occur in the form of deamidation, oxidation, disulfide bonds, cross‐linking, and protein aggregation (AOP #478) (Fochler and Durchschlag 1997). Additionally, there may be transcriptional, translational, or post‐translational modifications. Increases or decreases in protein expression, along with aggregation, may result in a tissue or organ level changes and further alterations in signaling.

In addition to protein modifications, DNA strand breaks result from ionization events. Ionizing radiation interacts directly with DNA, causing single‐strand breaks (SSBs), double‐strand breaks (DSBs), or clustered lesions (Rydberg 1996; Prise et al. 1999; Sutherland et al. 2000; Fakir et al. 2006; Timm et al. 2018). Indirectly, ROS generation may break DNA strands (AOP #478) (Mavragani et al. 2015; Nikjoo et al. 2016). These lesions may involve several types of damage to the DNA within a small area, typically 10 bp (Nikjoo et al. 2016). These lesions are caused by oxidation of the nitrogenous bases, with a particular propensity for guanine because of its low oxidation potential (Jovanovic and Simic 1986). The oxidation and damage to DNA may be detected within seconds to hours after radiation exposure (Bouten et al. 2021). The error‐prone repair mechanism of non‐homologous end joining (NHEJ) of clustered damage tends to produce non‐reversible DNA damage (Mavragani et al. 2015). Under normal conditions, the DNA may be repaired properly; however, during persistent oxidative stress, accumulation of large numbers of clustered lesions overloads the repair mechanisms, resulting in mutations such as insertions, deletions, and mismatches from incorrect repair (Turner 2002; Schoenfeld et al. 2012; Tangvarasittichai and Tangvarasittichai 2018). The damage may in parallel cause structural changes to the DNA backbone, alter its transcriptional access or replication, and create instability of the genome through chromosomal aberrations (Markkanen 2017). These molecular disruptions, if unresolved, cascade into broader tissue‐level consequences, where the accumulation of cellular damage and loss of cellular integrity may lead to alterations in tissue structure and function, ultimately affecting organ homeostasis as described below.

### 5.2 Tissue/Organ/Organism Level Events

Within the days to weeks following deposition of energy, the accumulated cellular damage that is not repaired contributes to a modification of tissue structure and function. Chronic inflammation over months to years may lead to sustained damage and a dysregulated cellular environment that contributes to age‐related phenotypes. Depending on the site of damage and the cell type, different adversities may ensue. Such pathologies may lead to many diseases, and the four target AOs examined in the AOP network include tissue/organ level effects on the bone, brain, eye, and vasculature. As described above, these pathways share common macromolecular and cellular‐level KEs, such as oxidative stress, DNA damage, and altered signaling. However, their mechanisms diverge into tissue and organ specific events, as outlined below.

In the context of the bone loss AOP, unrepaired damage to DNA, proteins, and lipids induced by free radicals may trigger cell death. Chronic oxidative stress and inflammation further disrupt cellular homeostasis, leading to the activation of apoptotic pathways, often via mitochondrial dysfunction. Both apoptosis (programmed cell death) and necrosis may occur within hours to days following exposure, depending on the extent of damage. For instance, ROS‐induced DNA damage may activate p53, which in turn promotes apoptosis (Ott et al. 2007). Oxidative stress is also associated with cell death through mitochondrial dysfunction, wherein calcium channels are opened, leading to the release of apoptotic factors and cellular swelling (Jilka et al. 2013; Memme et al. 2021; Sasi et al. 2015). Additionally, ROS may enhance autophagy by activating the transcription factor EB (TFEB)‐mediated signaling cascade (Johnson et al. 2020), thereby altering cellular signaling. TFEB regulates the autophagy‐lysosomal pathway, promoting the generation of autophagosomes under stressful conditions, such as when calcium is released from the lysosome (Song et al. 2021; Zhang et al. 2016). While autophagy is a normal physiological process in osteoblasts (Chatziravdeli et al. 2019; Guo et al. 2021), excessive autophagic flux may lead to cell death (Li et al. 2020). When combined with osteoclastic resorption and osteoblastic ossification, cell death exacerbates bone cell dysfunction. Disrupted signaling in osteoblasts and osteoclasts leads to dysregulated bone cell homeostasis, favoring bone resorption through an increased RANK‐L/OPG ratio, which stimulates osteoclastogenesis (Chatziravdeli et al. 2019; Jilka et al. 2013; Komori 2013). Furthermore, osteocyte apoptosis results in the release of pro‐osteoclastogenic factors, further promoting osteoclastogenesis and enhancing bone resorption (Komori 2013). At the same time, osteoblasts may undergo apoptosis or autophagy in response to ionizing damage, which reduces their activity and impairs bone formation (Wang et al. 2020; Xiong and O'Brien 2012). These alterations in osteoblast and osteoclast function disrupt bone homeostasis and remodeling, ultimately leading to a loss of bone matrix into the bloodstream and/or a reduction in bone deposition, culminating in bone loss (AO) (Donaubauer et al. 2020; Smith 2020; Willey et al. 2011).

For learning and memory impairment, ROS and increased DNA strand breaks may alter signaling pathways like MAPK or promote neuroinflammatory environments, causing neural remodeling. The signaling cascades lead to the expression of pro‐inflammatory cytokines, triggering an inflammatory response within hours. Adhesion molecules like intercellular adhesion molecule 1 (ICAM‐1) and vascular cell adhesion molecule 1 (VCAM‐1) may promote the entry of immune cells into tissues, where a chronic state of inflammation may induce senescence and cell death and impact neuronal morphology and neurogenesis in the brain (Tang et al. 2017; Wang et al. 2019). This encompasses various neuronal processes such as neurogenesis, neurodegeneration, and neuronal excitability, which impair learning and memory (AO) (Bálentová and Adamkov 2020; Monje and Palmer 2003). In addition, structural alterations such as demyelination, synaptic plasticity, and dendritic spine density may impair learning and memory (Donzis and Tronson 2014; Rachal Pugh et al. 2001; Yirmiya and Goshen 2011).

For the cataract AOP, deposition of ionizing radiation energy may cause DNA strand breaks, chromosomal aberrations, and changes to DNA structure, and an overall increase in mutation rate due to the cellular state of oxidative stress and poor repair mechanisms for DSBs. Such effects may alter oncogenes, which tend to promote proliferation when turned on/off by a mutation (Pitolli et al. 2019). For example, p53 is a tumor suppressor protein that arrests cells in the G1 phase (Chen et al. 2016). However, ionizing radiation at a high enough dose may cause irreparable DNA damage that inactivates p53, thereby causing uncontrolled cell proliferation (AOP #478) (Khan and Wang 2022). If this occurs in the lens of the eye, where there is little turnover, over time opacification will occur because of the rapid proliferation and incomplete differentiation in the germinative zone (GZ) of the lens epithelium (Ainsbury et al. 2016). In the GZ of the lens, rapid proliferation of lens epithelial cells may lead to incomplete differentiation of lens fiber cells. As these cells progress through the lens, they may retain organelles that would normally be lost during proper maturation. This failure to fully differentiate results in reduced lens transparency and contributes to the development of cataracts (AO) (Ainsbury et al. 2016; Hamada 2017; McCarron et al. 2022).

Oxidative stress in the vascular endothelium alters NO levels (AOP #470) in vascular endothelial cells. NO maintains vascular homeostasis by regulating vascular dilation, local cell growth, and platelet formation. ROS is able to uncouple eNOS (the enzyme that is used to synthesize NO from L‐arginine in endothelial cells) causing the production of more ROS, which convert NO to peroxynitrite, both of which reduce levels of available NO, causing endothelial dysfunction (AOP #470) (Förstermann 2010; Förstermann and Münzel 2006; Mitchell et al. 2019; Nagane et al. 2021; Soloviev and Kizub 2019). Indeed, lower cellular and serum levels of NO have been observed after different radiation exposures in many studies (Azimzadeh et al. 2015, 2017, 2021; Ait‐Aissa et al. 2022). Vascular endothelial cell dysfunction is associated with a lack of NO from oxidative stress, altered cell signaling, and prolonged inflammation from inflammatory mediators (Baran et al. 2021; Bonetti et al. 2003; Deanfield et al. 2007; Krüger‐Genge et al. 2019). This pathological activation of the endothelium generates senescent endothelial cells, which further contribute to disruption in vascular homeostasis along with phosphorylation of p53, leading to endothelial cell apoptosis (Borghini et al. 2013; Hughson et al. 2018; Schiffrin 2008; Senoner and Dichtl 2019; Soloviev and Kizub 2019; Venkatesulu et al. 2018). Abnormal vascular remodeling (AO) occurs in an effort to compensate for dysfunction. Some methods to combat non‐laminar flow and impaired repair include increasing vascular stiffness through thickening (Hsu et al. 2019), promoting leukocyte adhesion and fibrosis to aid repair (Sylvester et al. 2018), and enhancing arterial stiffness by increasing collagen and smooth muscle content (Zieman et al. 2005). Additionally, during periods of low NO, there is a reduction in elastin and extracellular matrix components (Hsu et al. 2019; Sylvester et al. 2018). These changes may culminate in accelerated atherosclerosis and a higher likelihood of thrombosis and embolisms (Boerma et al. 2016; Hughson et al. 2018; Slezak et al. 2017; Sylvester et al. 2018; Vernice et al. 2020).

Together, these KEs form the well‐established understanding of the mechanisms underlying this AOP network. The timeframe for these processes spans from immediate molecular damage to chronic tissue dysfunction and disease progression over months to years, illustrating the complex and prolonged impact of deposition of energy on biological systems.

## 6 Domain of Applicability

**FIGURE 5:**
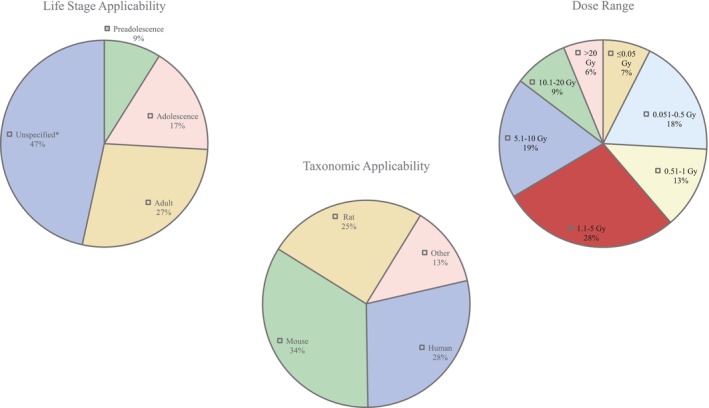
Summary of the domain of applicability. *Studies derived from in vitro models do not specify life stage.

The AOP network is applicable to vertebrates across all life stages and both sexes (Figure 5). The majority of the taxonomic evidence to support the causality of the network interactions is derived from studies in humans ( *Homo sapiens* ), human‐derived cell lines, mouse ( *Mus musculus* ), and rat ( *Rattus norvegicus* ) models, with less evidence coming from rhesus monkey ( *Macaca mulatta* ), rabbit ( *Oryctolagus cuniculus* ), and beagle dog ( *Canis lupus familiaris* ) models. The AOP is applicable to both sexes; however, most of the data to support the AOs were collected from male animals. This AOP network is relevant to all life stages; however, most available data (with specified lifestage) across species are derived from adult models. It should also be noted that bone loss, abnormal vascular remodeling, cataracts, and learning and memory impairment can be more prevalent in the middle‐aged population, and for most AOs, elderly populations were used in the evaluation of empirical studies.

## 7 Summary of Empirical Evidence

The modified Bradford‐Hill criteria of biological plausibility, essentiality of the KE, dose/incidence/temporal concordance, and quantitative understanding were used to evaluate the weight of evidence supporting the KERs and the AOPs.

Across the AOP network, the biological plausibility was considered strong for most KERs. The biological plausibility of the network was supported by extensive evidence demonstrating that most KERs are structurally and functionally well understood. The essentiality of most KEs was considered moderate, as a few knockout and transgenic animal models, as well as chemical modulators, provided insights into the roles of specific KEs at each step of the pathway. For example, studies elucidated how ROS‐induced DNA damage can lead to the formation of mutagenic lesions, which disrupt transcriptional and replicative processes, ultimately driving the pathway toward the AO (Liu et al. 2013; Turner et al. 2002). These structural insights are reinforced by functional studies demonstrating that the loss of key proteins, like RANK‐L in osteoblasts, disrupts bone remodeling and leads to exaggerated osteoclastogenesis, thereby impairing bone homeostasis (Chandra et al. 2014). Additionally, chemical modulation of autophagy pathways has revealed how dysregulated autophagic flux can result in cell death and tissue dysfunction, further supporting the functional relevance of these KEs in pathological states (Li et al. 2020; Wang et al. 2020). These findings provide moderate evidence that the individual KEs are not only mechanistically interlinked but also essential for the progression of AO. Collectively, the integration of structural and functional evidence strengthens the causal relationships within the pathway.

Most empirical evidence was derived from observational and experimental studies conducted using animal or in vitro models. While data from human space environment studies are limited, terrestrial radiation studies provided a substantial body of evidence. However, there was a lack of studies that utilized a broad range of doses and multiple time points, which are essential for effectively assessing the dose‐ and time‐dependence of KEs across the AOPs. The frequency of KEs was not often measured. KERs linked to deposition of energy tended to have stronger dose, time, and incidence support, due to more historical data originating from various radiation stressors. KERs from oxidative stress to the various KEs related to abnormal vascular remodeling and cataract AOs had less empirical evidence. Overall, the KER deposition of energy to oxidative stress contained a large body of consistent evidence to support dose‐ and time‐concordance across these KEs. Weakly supported KERs were predominantly adjacent KERs (e.g., altered signaling to increase endothelial dysfunction).

Due to the lack of strong empirical evidence for adjacent KERs, quantitative understanding of the AOP network is generally low. This is partly due to the lack of standardized reporting for data and assays, which complicates the ability to draw clear connections between upstream KEs and downstream effects. Moreover, many of the KERs are multifaceted, with numerous parameters and modulating factors (see below) contributing to the complex relationships. Currently, mathematical models do not exist that can reliably predict downstream effects based on upstream events; this could be an area of future research focus.

**FIGURE 6:**
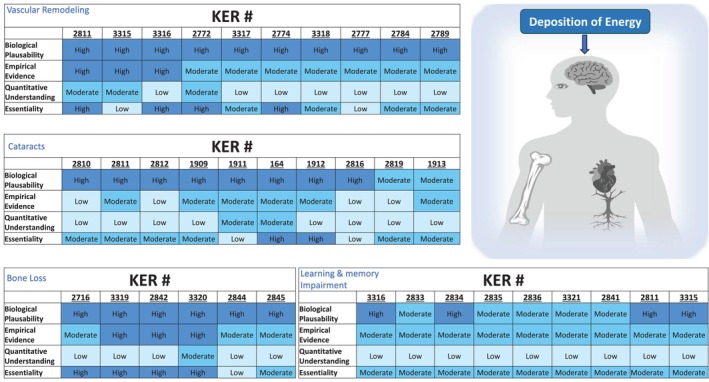
A summary of the evidence evaluation of adjacent key event relationships in the four adverse outcome pathways of cataracts, abnormal vascular remodeling, learning and memory impairment, and bone loss.

Some conflicting results have been observed; largely due to variability in study design, including differences in dose, dose rates, time points, radiation quality, experimental models, and clinical endpoints (e.g., incipient vs. mature cataracts). These factors should be considered in future studies to enhance the consistency and robustness of the empirical evidence, as well as to improve the quantitative understanding of the AOP network. A summary of the empirical evidence can be found in Figure 6.

## 8 Modulators

The AOP network can be modulated by a variety of factors such as age, sex, drugs, and genetics among others. Hormonal changes associated with aging can influence the risk of developing various diseases. Estrogen levels decrease with age, which can lead to decreased bone formation and increased bone resorption (Pacheco and Stock 2013; Willey et al. 2011). Furthermore, hormonal changes can cause the loss of elasticity in blood vessels and a build‐up of plaque that is associated with structural and functional changes to the endothelium. Accumulated damage from decreased DNA repair capacity with age increases the risk of CVD (Olivieri et al. 2013; North and Sinclair 2012; Vitale et al. 2009). Although men have a higher incidence of CVD than women, the risk is increased in post‐menopausal women (Garcia et al. 2016; Vitale et al. 2009). Additionally, some studies have shown that radiation‐induced cardiovascular events and mortality are significantly higher in women than men following radiotherapy (Khalid et al. 2020). Other studies suggest that females may be more susceptible to radiation‐induced cataracts than men (Azizova et al. 2018); however, more research is needed to fully understand this relationship (Barnard and Hamada 2023). In terms of brain disorders, a study in a mouse model of simulated GCR exposure showed that male mice, but not female mice, developed cognitive deficits, microglia activation, and synapse loss (Krukowski et al. 2018). Again, more research is needed to explore these sex differences, particularly due to the historical bias toward the more frequent use of male rodents than female ones, for various reasons (Mauvais‐Jarvis et al. 2017), which has led to knowledge gaps surrounding the female system.

Certain drugs modulate KERs in the AOP to bone loss, abnormal vascular remodeling, cataracts, and learning and memory impairment. For example, osteoclast inhibitors and osteoblast activators have a preventative effect against bone loss (Wissing 2015; Chandra et al. 2014; Yoshida et al. 2002), while some xenobiotics accelerate ionizing radiation‐induced bone loss (Rana et al. 2012). Anti‐inflammatory medications such as aspirin are potentially preventive against the development of radiation‐associated CVD (Camara Planek et al. 2020). Additionally, anti‐inflammatory agents reduce activated microglia and prevent learning and memory impairment (Monje and Palmer 2003; Greene‐Schloesser and Robbins 2012). Antioxidants also reduce levels of oxidative stress and subsequent disease progression (Domazetovic et al. 2017; Ostman et al. 2009; Manda et al. 2007; Thiagarajan and Manikandan 2013). The most well‐studied modulators of oxidative stress include: sulforaphane, epigallocatechin‐3‐gallate (EGCG), gallocatechin gallate (EGCG epimer), edaravone, fucoxanthin, nitroxides, thiols, and diphenyliodonium.

Another modulator of the AOP is genetics (Hamada and Fujimichi 2015; Barnard and Hamada 2023). Mutations in genes regulating bone metabolism, calcium absorption, and vitamin D metabolism are implicated in modulating bone loss (Mitchell and Yerges‐Armstrong 2011; Ranganathan 2009; Bai et al. 2020). There are several genomic loci associated with heart disease (Nelson et al. 2017; Nikpay et al. 2015). Sex‐specific genetic risk factors related to vascular remodeling can also contribute to CVD modulation (Berk and Korshunov 2006). Additionally, genes associated with lipid metabolism such as apolipoprotein E4 (ApoE4) variants are associated with an increased risk of CVDs (Mahley 2016). ApoE4 is also associated with an increased risk of developing Alzheimer's disease (Hunsberger et al. 2019; Zhu et al. 2012). Certain genetic polymorphisms related to DNA repair processes and antioxidant defense mechanisms may increase the risk of radiation‐induced cognitive impairment (Bucholtz and Demuth 2013).

Lifestyle factors, particularly diet, smoking, physical activity, and pre‐existing diseases can strongly influence the risk of a number of diseases (Mozaffarian et al. 2008; Turner et al. 2002; Little et al. 2024; Richardson 2022; Richardson et al. 2020). For example, physical activity and nutrition have been shown to prevent bone loss (Sibonga et al. 2019; Ackerman et al. 2021). Other examples are detailed in the AOP‐Wiki.

Overall, this proposed AOP network can be influenced by these modulators, which can alter the biological response to stressors. Understanding how these factors modify KEs within an AOP is an area for further exploration and is necessary for developing quantitative relationships.

## 9 Challenges and Knowledge Gaps

The development of this AOP network uncovered several uncertainties and knowledge gaps, with the major ones described here.

### 9.1 Inconsistent Findings on Measurement Levels

Some of the observed inconsistencies stem from the lack of clear, objective definitions and standardized measurements for certain KEs. For instance, the absence of a well‐defined endpoint for cataracts vs. lens opacities complicates literature searches and the establishment of accurate KERs. Similarly, defining broad endpoints such as CVD is challenging due to its complex etiology, which led to the adoption of a more specific endpoint: abnormal vascular remodeling. The KE “abnormal neuronal remodeling” also presented challenges during AOP development, as it encompasses a wide range of endpoints. In the case of NO levels, inconsistencies may arise from the diverse proxy measures used for its detection, as direct measurement of NO is difficult to conduct (Luiking et al. 2010). Further standardization in NO measurement could help refine the AOP to specify that this KE is linked to NO depletion.

### 9.2 Lack of Female‐Specific Mechanistic Data

Many KERs lack female‐specific mechanistic data. Typically, male models were studied, with females being less represented throughout the entire network. Human spaceflight studies examining sex differences tend to have low sample sizes because women make up less than 20% of astronauts (Lang et al. 2017). Some animal model studies that did examine both sexes did not separate male from female results (Chen et al. 2005; Lee et al. 2020; Russell et al. 2009).

### 9.3 Age‐Related Effects

Few mechanistic studies investigate the impact of age‐related effects on the four non‐cancer outcomes after radiation exposure.

### 9.4 Chronic and Low‐Dose Exposure Data

There is a relative lack of chronic and low‐dose (< 0.5 Gy (EPRI 2021)) exposure data, and several studies found unexpected or inconsistent effects of low dose radiation. There are currently efforts to create an experimental radiation field exposure scenario with more similarity to space (Norbury et al. 2016; Huff et al. 2023), which will be important in strengthening this AOP network.

### 9.5 Dose‐Concordance Discrepancies

Inconsistencies in dose‐concordance data between many KERs such as oxidative stress and pro‐inflammatory mediators complicate the understanding of their interplay.

### 9.6 Multiple Stressors

There are insufficient studies examining the combined effects of multiple space stressors on the bone, heart, eye, and brain. There is a need for more data relevant to space radiation (e.g., heavy ions, alpha particles, neutrons).

### 9.7 Qualitative Nature of the AOP

The proposed AOP network is qualitative and includes various radiation types and doses, necessitating an eventual quantitative understanding of dose, dose rate, and radiation quality effects on KEs and KERs.

### 9.8 Standardization of Measurement

There is a need for standardized methods to measure and interpret endpoints to refine the AOP and specify the KE.

### 9.9 Consistent Data Reporting

Prioritization is required for experiments that use the same experimental platform to assess multiple KEs in an AOP. This will allow comparison of results and facilitate the understanding of time concordance and dose concordance effects. Consistent data reporting is needed to validate quantitative aspects of the relationships between KERs.

### 9.10 Relevant Models

KERs lacked evidence from models that were relevant to the respective AO, which gave poor inferential strength to the KER. For example, there were no lens‐specific data for some KERs (e.g., inadequate DNA repair to increase mutations; oxidative DNA damage to inadequate DNA repair; increase DNA strand breaks to inadequate DNA repair; inadequate repair to chromosomal aberrations; increase mutations to cell proliferation; chromosomal aberration to increase cell proliferation) within the cataracts pathway, making it unclear how these KEs contribute specifically to cataracts. Alternatively, some KERs (e.g., oxidative stress to DNA strand breaks; pro‐inflammatory mediators to abnormal neural remodeling) within the impaired learning and memory pathway had minimal evidence from human studies to evaluate the modified Bradford‐Hill criteria.

### 9.11 Adaptive Responses

Adaptive responses have also been observed in animal and in vitro models, where low doses of radiation can induce a protective effect (reviewed in Guéguen et al. 2019); but its implications for human health effects are not clear.

## 10 Actionable Recommendations

To fully leverage the AOP framework for risk assessment purposes, we recommend key steps that must be taken to improve the quantitative understanding of the AOP network through new experimental research (Table 2):

- *Select and measure more than one KER in a study*: Design and conduct experiments that identify and quantify at least two KERs at different stages of the AOP (e.g., molecular, cellular, tissue‐level, organism‐level). These should capture early damage responses (e.g., DNA damage, oxidative stress) and link to downstream phenotypic effects (e.g., inflammation, functional/behavioral impairment). Including multiple KEs in experiments is essential to understanding both the qualitative and quantitative relationships across the AOPs.

**TABLE 2:** Recommended models and endpoints across biological levels for selected adverse outcome pathways (AOPs).

Adverse outcome (AO)	Key event relationship (KER)	Relevant cell types	Best models	Molecular‐level endpoints	Cellular‐level endpoints	Organism‐level endpoints
Bone loss	Oxidative stress to Oxidative DNA damage to Bone cell homeostasis to bone remodeling (Osteoblast/osteoclast disruption leading to bone resorption)	Osteoblasts Osteoclasts Bone marrow stromal cells (BMSCs)	3D organoid model of bone tissue Scaffold‐based co‐culture of osteoblasts and osteoclasts	DNA double‐strand breaks (γ‐H2AX) Mitochondrial dysfunction (ATP levels) Oxidative damage markers (8‐OHdG)	Osteoclast differentiation markers (RANKL, OPG) Calcium resorption Inflammatory cytokines (IL‐6, TNF)	Mouse or Rat: Bone mineral density (DXA), trabecular microarchitecture (micro‐CT), mechanical strength (three‐point bending)
Abnormal vascular remodeling	Endothelial dysfunction to abnormal vascular remodeling (Endothelial cell injury leading to increased arterial stiffness or plaque formation)	Endothelial cells Smooth muscle cells Pericytes	3D endothelial/vascular tissue‐on‐chip Perfusable vascular networks with endothelial and smooth muscle cells	Endothelial DNA damage (γ‐H2AX) Lipid peroxidation (MDA) Nitric oxide (NO) signaling disruption	Oxidative stress markers (ROS) Endothelial dysfunction (VEGF, eNOS) Smooth muscle proliferation Vascular stiffness (elastin/collagen ratio)	ApoE−/− or LDLR−/− Mice: Aortic plaque size (histology), arterial stiffness (pulse wave velocity)
Learning and memory impairment	Oxidative stress to proinflammatory mediators to neural remodeling (Neuronal oxidative stress leading to neuroinflammation and synaptic dysfunction)	Neurons Astrocytes Microglia Pericyte Blood–brain barrier endothelial cells	3D cerebral organoids Neuron‐astrocyte co‐culture models Blood–brain barrier‐on‐chip	Lipid peroxidation (MDA) Oxidative DNA damage (8‐OHdG) Protein oxidation (carbonyl content)	Neuroinflammatory markers (IL‐1β, TNF‐α) Synaptic proteins (PSD‐95) Neuronal long‐term potentiation	Mouse or Rat: Morris water maze (spatial memory), novel object recognition (learning), fear conditioning (emotional memory, discrimination learning)
Cataracts	Oxidative stress to protein modification (Lens epithelial macromolecular damage leading to opacity)	Lens epithelial cells Lens fibers	Lens‐on‐chip models 3D lens organoids	Oxidative stress markers (SOD, GPx) DNA damage markers (γ‐H2AX) Lipid peroxidation (4‐HNE, MDA)	Protein cross‐linking (lens crystallins) Protein aggregation Apoptosis markers (Caspase‐3, BAX) Lens transparency (functional marker of cataract formation)	Mouse/Rat/Rabbit: Slit lamp exam for lens opacity, Scheimpflug imaging, histopathology

*Note*: This table summarizes key experimental models and endpoints considered relevant for AOP development. It is not exhaustive and should be adapted to specific research contexts.

Abbreviations: 4‐HNE, 4‐hydroxy‐2‐nonenal; 8‐OHdG, 8‐hydroxy‐2′‐deoxyguanosine; ATP, adenosine triphosphate; BAX, Bcl‐2 associated X‐protein; BMSC, bone marrow stromal cell; eNOS, endothelial nitric oxide synthase; GPx, glutathione peroxidase; IL‐1β, interleukin‐1β; IL‐6, Interleukin 6; KER, key event relationship; MDA, malondialdehyde; NO, nitric oxide; OPG, osteoprotegerin; PSD‐95, postsynaptic density protein 95; RANKL, receptor activator of nuclear factor‐kappa B ligand; ROS, reactive oxygen species; SOD, superoxide dismutase; TNF, tumor necrosis factor; TNF‐α, tumor necrosis factor α; VEGF, vascular endothelial growth factor; γ‐H2AX, the phosphorylated form (γ) of the histone H2AX.

### 10.1 Use Both In Vitro and In Vivo Models

- Employ relevant 2D/3D in vitro models (e.g., organoids, organ‐on‐chip) that reflect tissue‐specific responses and allow for high‐throughput mechanistic investigations. In parallel, utilize appropriate animal models to evaluate systemic and higher‐order effects that cannot be captured in vitro, such as learning and memory. Selection of the species should be guided by the specific AOs under investigation and the suitability of the model for measuring relevant AO‐specific phenotypic endpoints. *Use a Broad Dose Range and Multiple Timepoints*: Design experiments to cover a wide range of doses, including both low‐dose and high‐dose exposures relevant to space radiation. Conduct assessments at multiple time points to capture both early and late events along the AOP, enhancing temporal mapping of the AOP.
- *Assess Multiple Endpoints*: Measure macromolecular (e.g., DNA damage, oxidative stress) and cellular/functional endpoints (e.g., inflammation, cell viability, tissue remodeling, behavioral outcomes) aligned with KEs of the AOP to provide comprehensive data on the progression of radiation effects.
- *Incorporate Multi‐scale and Multi‐omic Technologies*: Use high‐throughput omics technologies (e.g., genomics, proteomics, metabolomics) to integrate molecular data with phenotypic outcomes. This will provide predictive mechanistic understanding of the dose–response relationships.
- *Apply Dose–Response Modeling Approaches*: Use quantitative models to analyze the data, including benchmark dose modeling and point of departure identification (Chauhan et al. 2023; Vuong et al. 2025). This step will help to define the dose at which molecular‐level damage progresses into observable AOs.

## 11 Recommended Cell Types to Understand the Cellular/Molecular Mechanisms Leading to Each AO

- *Bone Loss*: Osteoblasts (bone formation), osteoclasts (bone resorption), and bone marrow stromal cells (BMSCs) for their role in bone remodeling.
- *Vascular Remodeling*: Endothelial cells, smooth muscle cells, and fibroblasts as they form the vascular structure and are affected by radiation‐induced remodeling.
- *Learning and Memory Impairment*: Neurons, astrocytes (supporting cells), microglia (immune cells of the brain), brain pericytes, and blood–brain barrier endothelial cells, which are all involved in neuroinflammation and cognitive decline.
- *Cataracts*: Lens epithelial cells and lens fibers are central to cataract formation.

### 11.1 Radiation Type

Space‐relevant radiation types like heavy ions, neutrons, protons, and gamma radiation simulate exposure to GCR and SPEs. Consider well‐characterized low and high doses, variable dose rates, and mixed fields of particle types relevant to the space radiation environment.

### 11.2 Macromolecular Endpoints

Focus on early molecular events like DNA damage, oxidative damage 8‐hydroxy‐2′‐deoxyguanosine (8‐OHdG) and malondialdehyde (MDA), ATP depletion (mitochondrial dysfunction) which precede cellular changes and are critical to capturing early radiation effects.

### 11.3 Cellular/Functional Endpoints

Measure outcomes like persistent pro‐inflammatory mediators, calcium resorption, cell death (apoptosis, ferroptosis, necrosis) or synaptic dysfunction to correlate molecular damage with adverse physiological changes.

## 12 Conclusions and Future Direction

This AOP network has collated decades of research in the radiation field. The biological plausibility for the network is strong, grounded in mechanistic studies that demonstrate the functional progression from specific KEs to AOs, though some uncertainties and limitations remain, particularly concerning quantitative understanding. The network's applicability domain covers relevant taxonomies, life stages, and sexes, ensuring broad relevance across species and developmental stages. Various known space stressors trigger the AOPs, and existing dose and time‐response data across KERs provide a foundational understanding of how these stressors propagate biological damage. It is important to acknowledge that early KEs may be initiated, but repair mechanisms and adaptation processes can prevent the full progression to an AO. Progression to clinical diseases depends on various modulating factors, including dose, individual susceptibility, and interactions with other spaceflight‐related stressors like microgravity. Therefore, the likelihood of some early molecular disruptions evolving into severe, clinically observable diseases over the course of typical mission durations remains low. The advantage of employing the AOP framework is to identify specific KEs that may be modulated before they escalate into more significant AOs and to have a fully quantitative AOP that can be used to support risk model development.

To achieve this goal, future work may be directed to strengthening the KERs that currently have low evidence in the form of the modified Bradford‐Hill criteria and to developing studies using human‐relevant models (Figure 6). Some KEs (cellular senescence, epigenetic modifications, mitochondria dysfunction, miRNA expression) that were considered in the original network were ultimately excluded due to a lack of evidence supporting their inclusion (summarized in: Kozbenko et al. 2024; Carrothers et al. 2024; Sandhu et al. 2024; Sleiman et al. 2024). This can be addressed by directing more research toward these potential KEs to explore their roles in the pathway. The AOP network could also be strengthened by considering other factors that underlie the development of KEs to disease such as gene expression changes and epigenetics. Additionally, some of the current KEs are broad (e.g., neural remodeling, endothelial dysfunction) and encompass many different endpoints; these may be further refined in the future to gain a better quantitative understanding of the pathways. Development of standardized guidelines for reporting, evaluating, and presenting data would also benefit AOPs as consistent reporting would make interstudy comparisons between studies feasible. To improve the use of AOPs in the context of space research, future research would require studies using multiple space‐relevant radiation types with low‐dose chronic exposure in combination with microgravity.

Overall, the proposed AOP network helps to better characterize and assess risk by providing a structured framework to integrate mechanistic data, with the potential to become increasingly informative as quantitative relationships between KEs are developed. The risk characterization will help determine whether there is a need for risk management strategies. In addition, the collation of information provided by this AOP network will be relevant to the work of international radiation organizations such as the ICRP Task Groups related to space radiation (Task Group on Radiation Protection in Space et al. 2013), the US National Council on Radiation Protection and Measurements (NCRP) and the United Nations Scientific Committee on the Effects of Atomic Radiation (UNSCEAR), particularly as they review the scientific consensus that forms the basis of the system of radiological protection. Lastly, as we enter the next space age, marked by an influx of commercial space missions, such as Inspiration4 and Polaris Dawn, this expanding landscape provides a robust foundation for generating comprehensive data (Mason et al. 2024; Jones et al. 2024; Overbey et al. 2024). These data will be critical in building a complete AOP network to inform and guide future missions with effective risk management strategies.

## Conflicts of Interest

The authors declare no conflicts of interest.

## Data Availability

The data that support the findings of this study are available in aop wiki at https://www.aopwiki.org/. These data were derived from the following resources available in the public domain: https://www.aopwiki.org/, https://www.aopwiki.org/.

## References

[em70031-bib-0001] Ackerman, K. E. , K. L. Popp , and M. L. Bouxsein . 2021. “Rocket Science: What Spaceflight Can Tell Us About Skeletal Health on Earth.” British Journal of Sports Medicine 55, no. 21: 1182–1183. 33883169 10.1136/bjsports-2021-104164

[em70031-bib-0002] Afshinnekoo, E. , R. T. Scott , M. J. MacKay , et al. 2020. “Fundamental Biological Features of Spaceflight: Advancing the Field to Enable Deep‐Space Exploration.” Cell 183, no. 5: 1162–1184. 33242416 10.1016/j.cell.2020.10.050 PMC8441988

[em70031-bib-0003] Ainsbury, E. A. , S. Barnard , S. Bright , et al. 2016. “Ionizing Radiation Induced Cataracts: Recent Biological and Mechanistic Developments and Perspectives for Future Research.” Mutation Research, Reviews in Mutation Research 770, no. Pt B: 238–261. 27919334 10.1016/j.mrrev.2016.07.010

[em70031-bib-0004] Ait‐Aissa, K. , O. M. Koval , N. R. Lindsey , and I. M. Grumbach . 2022. “Mitochondrial Ca^2+^ Uptake Drives Endothelial Injury by Radiation Therapy.” Arteriosclerosis, Thrombosis, and Vascular Biology 42, no. 9: 1121–1136. 35899616 10.1161/ATVBAHA.122.317869 PMC9394506

[em70031-bib-0005] Ankley, G. T. , R. S. Bennett , R. J. Erickson , et al. 2010. “Adverse Outcome Pathways: A Conceptual Framework to Support Ecotoxicology Research and Risk Assessment.” Environmental Toxicology and Chemistry 29, no. 3: 730–741. 20821501 10.1002/etc.34

[em70031-bib-0006] Antonsen, E. L. , E. Connell , W. Anton , R. J. Reynolds , D. M. Buckland , and M. Van Baalen . 2023. “Updates to the NASA Human System Risk Management Process for Space Exploration.” NPJ Microgravity 9, no. 1: 72. 37679359 10.1038/s41526-023-00305-z PMC10485075

[em70031-bib-0008] Azimzadeh, O. , W. Sievert , H. Sarioglu , et al. 2015. “Integrative Proteomics and Targeted Transcriptomics Analyses in Cardiac Endothelial Cells Unravel Mechanisms of Long‐Term Radiation‐Induced Vascular Dysfunction.” Journal of Proteome Research 14, no. 2: 1203–1219. 25590149 10.1021/pr501141b

[em70031-bib-0009] Azimzadeh, O. , V. Subramanian , W. Sievert , et al. 2021. “Activation of PPARα by Fenofibrate Attenuates the Effect of Local Heart High Dose Irradiation on the Mouse Cardiac Proteome.” Biomedicine 9, no. 12: 1845. 10.3390/biomedicines9121845 PMC8698387 34944662

[em70031-bib-0010] Azimzadeh, O. , V. Subramanian , S. Ständer , et al. 2017. “Proteome Analysis of Irradiated Endothelial Cells Reveals Persistent Alteration in Protein Degradation and the RhoGDI and NO Signalling Pathways.” International Journal of Radiation Biology 93, no. 9: 920–928. 28697312 10.1080/09553002.2017.1339332

[em70031-bib-0011] Azizova, T. V. , M. V. Bannikova , E. S. Grigoryeva , V. L. Rybkina , and N. Hamada . 2020. “Occupational Exposure to Chronic Ionizing Radiation Increases Risk of Parkinson's Disease Incidence in Russian Mayak Workers.” International Journal of Epidemiology 49, no. 2: 435–447. 31722376 10.1093/ije/dyz230

[em70031-bib-0012] Azizova, T. V. , E. V. Bragin , N. Hamada , and M. V. Bannikova . 2016. “Risk of Cataract Incidence in a Cohort of Mayak PA Workers Following Chronic Occupational Radiation Exposure.” PLoS One 11, no. 10: e0164357. 27723789 10.1371/journal.pone.0164357 PMC5056693

[em70031-bib-0013] Azizova, T. V. , N. Hamada , E. S. Grigoryeva , and E. V. Bragin . 2018. “Risk of Various Types of Cataracts in a Cohort of Mayak Workers Following Chronic Occupational Exposure to Ionizing Radiation.” European Journal of Epidemiology 33, no. 12: 1193–1204. 30306422 10.1007/s10654-018-0450-4

[em70031-bib-0014] Bai, J. , Y. Wang , J. Wang , J. Zhai , F. He , and G. Zhu . 2020. “Irradiation‐Induced Senescence of Bone Marrow Mesenchymal Stem Cells Aggravates Osteogenic Differentiation Dysfunction via Paracrine Signaling.” American Journal of Physiology. Cell Physiology 318, no. 5: C1005–C1017. 32233952 10.1152/ajpcell.00520.2019

[em70031-bib-0015] Bálentová, S. , and M. Adamkov . 2020. “Pathological Changes in the Central Nervous System Following Exposure to Ionizing Radiation.” Physiological Research 69, no. 3: 389–404. 32469226 10.33549/physiolres.934309 PMC8648310

[em70031-bib-0016] Baran, R. , S. Marchal , S. Garcia Campos , et al. 2021. “The Cardiovascular System in Space: Focus on In Vivo and In Vitro Studies.” Biomedicine 10, no. 1: 59. 10.3390/biomedicines10010059 PMC8773383 35052739

[em70031-bib-0017] Barnard, S. G. R. , and N. Hamada . 2023. “Individual Response of the Ocular Lens to Ionizing Radiation.” International Journal of Radiation Biology 99, no. 2: 138–154. 35536112 10.1080/09553002.2022.2074166

[em70031-bib-0018] Baxter, N. N. , E. B. Habermann , J. E. Tepper , S. B. Durham , and B. A. Virnig . 2005. “Risk of Pelvic Fractures in Older Women Following Pelvic Irradiation.” Journal of the American Medical Association 294, no. 20: 2587–2593. 16304072 10.1001/jama.294.20.2587

[em70031-bib-0019] Becker, R. A. , G. T. Ankley , S. W. Edwards , et al. 2015. “Increasing Scientific Confidence in Adverse Outcome Pathways: Application of Tailored Bradford‐Hill Considerations for Evaluating Weight of Evidence.” Regulatory Toxicology and Pharmacology 72, no. 3: 514–537. 25863193 10.1016/j.yrtph.2015.04.004

[em70031-bib-0020] Berk, B. C. , and V. A. Korshunov . 2006. “Genetic Determinants of Vascular Remodelling.” Canadian Journal of Cardiology 22, no. Suppl B: 6B–11B. 10.1016/s0828-282x(06)70980-1 PMC2780836 16498506

[em70031-bib-0021] Betlazar, C. , R. J. Middleton , R. B. Banati , and G. J. Liu . 2016. “The Impact of High and Low Dose Ionising Radiation on the Central Nervous System.” Redox Biology 9: 144–156. 27544883 10.1016/j.redox.2016.08.002 PMC4993858

[em70031-bib-0022] Boerma, M. , V. Sridharan , X. W. Mao , et al. 2016. “Effects of Ionizing Radiation on the Heart.” Mutation Research, Reviews in Mutation Research 770, no. Pt B: 319–327. 27919338 10.1016/j.mrrev.2016.07.003 PMC5144922

[em70031-bib-0023] Bonetti, P. O. , L. O. Lerman , and A. Lerman . 2003. “Endothelial Dysfunction: A Marker of Atherosclerotic Risk.” Arteriosclerosis, Thrombosis, and Vascular Biology 23, no. 2: 168–175. 12588755 10.1161/01.atv.0000051384.43104.fc

[em70031-bib-0024] Borghini, A. , E. A. Gianicolo , E. Picano , and M. G. Andreassi . 2013. “Ionizing Radiation and Atherosclerosis: Current Knowledge and Future Challenges.” Atherosclerosis 230, no. 1: 40–47. 23958250 10.1016/j.atherosclerosis.2013.06.010

[em70031-bib-0025] Bouten, R. M. , E. F. Young , R. Selwyn , D. Iacono , W. B. Rittase , and R. M. Day . 2021. “Tissue Barriers in Disease, Injury and Regeneration.” In Effects of Radiation on Endothelial Barrier and Vascular Integrity, 43–94. Elsevier.

[em70031-bib-0026] Bucholtz, N. , and I. Demuth . 2013. “DNA‐Repair in Mild Cognitive Impairment and Alzheimer's Disease.” DNA Repair 12, no. 10: 811–816. 23919922 10.1016/j.dnarep.2013.07.005

[em70031-bib-0027] Camara Planek, M. I. , A. J. Silver , A. S. Volgman , and T. M. Okwuosa . 2020. “Exploratory Review of the Role of Statins, Colchicine, and Aspirin for the Prevention of Radiation‐Associated Cardiovascular Disease and Mortality.” Journal of the American Heart Association 9, no. 2: e014668. 31960749 10.1161/JAHA.119.014668 PMC7033839

[em70031-bib-0028] Carrothers, E. , M. Appleby , V. Lai , et al. 2024. “AOP Report: Development of an Adverse Outcome Pathway for Deposition of Energy Leading to Cataracts.” Environmental and Molecular Mutagenesis 65, no. S3: 31–56. 38644659 10.1002/em.22594

[em70031-bib-0029] Cekanaviciute, E. , S. Rosi , and S. V. Costes . 2018. “Central Nervous System Responses to Simulated Galactic Cosmic Rays.” International Journal of Molecular Sciences 19, no. 11: 3669. 30463349 10.3390/ijms19113669 PMC6275046

[em70031-bib-0030] Chancellor, J. C. , G. B. Scott , and J. P. Sutton . 2014. “Space Radiation: The Number One Risk to Astronaut Health Beyond Low Earth Orbit.” Life 4, no. 3: 491–510. 25370382 10.3390/life4030491 PMC4206856

[em70031-bib-0031] Chandra, A. , T. Lin , M. B. Tribble , et al. 2014. “PTH1‐34 Alleviates Radiotherapy‐Induced Local Bone Loss by Improving Osteoblast and Osteocyte Survival.” Bone 67: 33–40. 24998454 10.1016/j.bone.2014.06.030 PMC4154509

[em70031-bib-0032] Chang, H. H. Y. , N. R. Pannunzio , N. Adachi , and M. R. Lieber . 2017. “Non‐Homologous DNA End Joining and Alternative Pathways to Double‐Strand Break Repair.” Nature Reviews. Molecular Cell Biology 18, no. 8: 495–506. 28512351 10.1038/nrm.2017.48 PMC7062608

[em70031-bib-0033] Chatziravdeli, V. , G. N. Katsaras , and G. I. Lambrou . 2019. “Gene Expression in Osteoblasts andOsteoclasts Under Microgravity Conditions: A Systematic Review.” Current Genomics 20, no. 3: 184–198. 31929726 10.2174/1389202920666190422142053 PMC6935951

[em70031-bib-0034] Chauhan, V. , D. Beaton , N. Hamada , et al. 2022. “Adverse Outcome Pathway: A Path Toward Better Data Consolidation and Global Co‐Ordination of Radiation Research.” International Journal of Radiation Biology 98, no. 12: 1694–1703. 34919011 10.1080/09553002.2021.2020363

[em70031-bib-0035] Chauhan, V. , D. Beaton , K. E. Tollefsen , et al. 2024. “Radiation Adverse Outcome Pathways (AOPs): Examining Priority Questions From an International Horizon‐Style Exercise.” International Journal of Radiation Biology 100, no. 7: 982–995. 38718325 10.1080/09553002.2024.2348072

[em70031-bib-0036] Chauhan, V. , N. Hamada , J. Garnier‐Laplace , et al. 2022. “Establishing a Communication and Engagement Strategy to Facilitate the Adoption of the Adverse Outcome Pathways in Radiation Research and Regulation.” International Journal of Radiation Biology 98, no. 12: 1714–1721. 35666945 10.1080/09553002.2022.2086716

[em70031-bib-0037] Chauhan, V. , Z. Said , J. Daka , et al. 2019. “Is There a Role for the Adverse Outcome Pathway Framework to Support Radiation Protection?” International Journal of Radiation Biology 95, no. 2: 225–232. 30373433 10.1080/09553002.2019.1532617

[em70031-bib-0038] Chauhan, V. , S. Sherman , Z. Said , C. L. Yauk , and R. Stainforth . 2021. “A Case Example of a Radiation‐Relevant Adverse Outcome Pathway to Lung Cancer.” International Journal of Radiation Biology 97, no. 1: 68–84. 31846388 10.1080/09553002.2019.1704913

[em70031-bib-0039] Chauhan, V. , J. Yu , N. Vuong , et al. 2023. “Considerations for Application of Benchmark Dose Modeling in Radiation Research: Workshop Highlights.” International Journal of Radiation Biology 99, no. 9: 1320–1331. 36881459 10.1080/09553002.2023.2181998

[em70031-bib-0040] Chen, H. , Z. Z. Chong , S. M. De Toledo , E. I. Azzam , S. Elkabes , and N. Souayah . 2016. “Delayed Activation of Human Microglial Cells by High Dose Ionizing Radiation.” Brain Research 1646: 193–198. 27265419 10.1016/j.brainres.2016.06.002

[em70031-bib-0041] Chen, Y. , O. Hyrien , J. Williams , P. Okunieff , T. Smudzin , and P. Rubin . 2005. “Interleukin (IL)‐1A and IL‐6: Applications to the Predictive Diagnostic Testing of Radiation Pneumonitis.” International Journal of Radiation Oncology, Biology, Physics 62, no. 1: 260–266. 15850931 10.1016/j.ijrobp.2005.01.041

[em70031-bib-0042] Chylack, L. T., Jr. , A. H. Feiveson , L. E. Peterson , et al. 2012. “NASCA Report 2: Longitudinal Study of Relationship of Exposure to Space Radiation and Risk of Lens Opacity.” Radiation Research 178, no. 1: 25–32. 22687051 10.1667/rr2876.1

[em70031-bib-0043] Chylack, L. T., Jr. , L. E. Peterson , A. H. Feiveson , et al. 2009. “NASA Study of Cataract in Astronauts (NASCA). Report 1: Cross‐Sectional Study of the Relationship of Exposure to Space Radiation and Risk of Lens Opacity.” Radiation Research 172, no. 1: 10–20. 19580503 10.1667/RR1580.1

[em70031-bib-0044] Collett, G. , K. Craenen , W. Young , M. Gilhooly , and R. M. Anderson . 2020. “The Psychological Consequences of (Perceived) Ionizing Radiation Exposure: A Review on Its Role in Radiation‐Induced Cognitive Dysfunction.” International Journal of Radiation Biology 96, no. 9: 1104–1118. 32716221 10.1080/09553002.2020.1793017

[em70031-bib-0045] Cucinotta, F. A. , M. Alp , F. M. Sulzman , and M. Wang . 2014. “Space Radiation Risks to the Central Nervous System.” Life Sciences in Space Research 2: 54–69.

[em70031-bib-0046] Deanfield, J. E. , J. P. Halcox , and T. J. Rabelink . 2007. “Endothelial Function and Dysfunction: Testing and Clinical Relevance.” Circulation 115, no. 10: 1285–1295. 17353456 10.1161/CIRCULATIONAHA.106.652859

[em70031-bib-0047] Desouky, O. , N. Ding , and G. Zhou . 2015. “Targeted and Non‐Targeted Effects of Ionizing Radiation.” Journal of Radiation Research and Applied Sciences 8, no. 2: 247–254.

[em70031-bib-0048] Domazetovic, V. , G. Marcucci , T. Iantomasi , M. L. Brandi , and M. T. Vincenzini . 2017. “Oxidative Stress in Bone Remodeling: Role of Antioxidants.” Clinical Cases in Mineral and Bone Metabolism: The Official Journal of the Italian Society of Osteoporosis, Mineral Metabolism, and Skeletal Diseases 14, no. 2: 209–216. 29263736 10.11138/ccmbm/2017.14.1.209 PMC5726212

[em70031-bib-0049] Donaubauer, A. J. , L. Deloch , I. Becker , R. Fietkau , B. Frey , and U. S. Gaipl . 2020. “The Influence of Radiation on Bone and Bone Cells‐Differential Effects on Osteoclasts and Osteoblasts.” International Journal of Molecular Sciences 21, no. 17: 6377. 32887421 10.3390/ijms21176377 PMC7504528

[em70031-bib-0050] Donzis, E. J. , and N. C. Tronson . 2014. “Modulation of Learning and Memory by Cytokines: Signaling Mechanisms and Long Term Consequences.” Neurobiology of Learning and Memory 115: 68–77. 25151944 10.1016/j.nlm.2014.08.008 PMC4250287

[em70031-bib-0051] Elsevier . 2017. “Guidance Notes for Authors of Systematic Reviews, Systematic Evidence Mapsand Related Manuscript Types.” Elsevier. https://www.elsevier.com/journals/environmentinternational/0160‐4120/guidance‐notes.

[em70031-bib-0052] EPRI . 2021. “Electric Power Research Institute (EPRI), the International Dose Effect Alliance (IDEA) Virtual Workshop – November 2021.” https://www.epri.com/research/programs/061197/events/F87E2EBE‐CDC8‐415F‐9279‐F322E6E54326.

[em70031-bib-0053] Fakir, H. , R. K. Sachs , B. Stenerlöw , and W. Hofmann . 2006. “Clusters of DNA Double‐Strand Breaks Induced by Different Doses of Nitrogen Ions for Various LETs: Experimental Measurements and Theoretical Analyses.” Radiation Research 166, no. 6: 917–927. 17149976 10.1667/RR0639.1

[em70031-bib-0054] Fochler, C. , and H. Durchschlag . 1997. “Investigation of Irradiated Eye‐Lens Proteins by Analytical Ultracentrifugation and Other Techniques.” Progress in Colloid and Polymer Science 107: 94–101.

[em70031-bib-0055] Förstermann, U. 2010. “Nitric Oxide and Oxidative Stress in Vascular Disease.” Pflügers Archiv: European Journal of Physiology 459, no. 6: 923–939. 20306272 10.1007/s00424-010-0808-2

[em70031-bib-0056] Förstermann, U. , and T. Münzel . 2006. “Endothelial Nitric Oxide Synthase in Vascular Disease: From Marvel to Menace.” Circulation 113, no. 13: 1708–1714. 16585403 10.1161/CIRCULATIONAHA.105.602532

[em70031-bib-0057] Garcia, M. , S. L. Mulvagh , C. N. Merz , J. E. Buring , and J. E. Manson . 2016. “Cardiovascular Disease in Women: Clinical Perspectives.” Circulation Research 118, no. 8: 1273–1293. 27081110 10.1161/CIRCRESAHA.116.307547 PMC4834856

[em70031-bib-0058] Gillies, M. , D. B. Richardson , E. Cardis , et al. 2017. “Mortality From Circulatory Diseases and Other Non‐Cancer Outcomes Among Nuclear Workers in France, the United Kingdom and the United States (INWORKS).” Radiation Research 188, no. 3: 276–290. 28692406 10.1667/RR14608.1 PMC5651512

[em70031-bib-0059] Greene‐Schloesser, D. , and M. E. Robbins . 2012. “Radiation‐Induced Cognitive Impairment – From Bench to Bedside.” Neuro‐Oncology 14, no. S4: 37–44. 10.1093/neuonc/nos196 PMC3480242 23095829

[em70031-bib-0060] Guéguen, Y. , A. Bontemps , and T. G. Ebrahimian . 2019. “Adaptive Responses to Low Doses of Radiation or Chemicals: Their Cellular and Molecular Mechanisms.” Cellular and Molecular Life Sciences: CMLS 76, no. 7: 1255–1273. 30535789 10.1007/s00018-018-2987-5 PMC11105647

[em70031-bib-0061] Guo, Y. F. , T. Su , M. Yang , et al. 2021. “The Role of Autophagy in Bone Homeostasis.” Journal of Cellular Physiology 236, no. 6: 4152–4173. 33452680 10.1002/jcp.30111

[em70031-bib-0062] Hamada, N. 2017. “Ionizing Radiation Sensitivity of the Ocular Lens and Its Dose Rate Dependence.” International Journal of Radiation Biology 93, no. 10: 1024–1034. 27899034 10.1080/09553002.2016.1266407

[em70031-bib-0063] Hamada, N. , T. V. Azizova , and M. P. Little . 2020. “An Update on Effects of Ionizing Radiation Exposure on the Eye.” British Journal of Radiology 93, no. 1115: 20190829. 31670577 10.1259/bjr.20190829 PMC8519632

[em70031-bib-0064] Hamada, N. , and Y. Fujimichi . 2015. “Role of Carcinogenesis Related Mechanisms in Cataractogenesis and Its Implications for Ionizing Radiation Cataractogenesis.” Cancer Letters 368, no. 2: 262–274. 25687882 10.1016/j.canlet.2015.02.017

[em70031-bib-0065] Hamada, N. , K. I. Kawano , F. M. Yusoff , et al. 2020. “Ionizing Irradiation Induces Vascular Damage in the Aorta of Wild‐Type Mice.” Cancers 12, no. 10: 3030. 33081026 10.3390/cancers12103030 PMC7603246

[em70031-bib-0066] Hamada, N. , M. Maeda , K. Otsuka , and M. Tomita . 2011. “Signaling Pathways Underpinning the Manifestations of Ionizing Radiation‐Induced Bystander Effects.” Current Molecular Pharmacology 4, no. 2: 79–95. 21143186 10.2174/1874467211104020079

[em70031-bib-0067] Hamada, N. , H. Matsumoto , T. Hara , and Y. Kobayashi . 2007. “Intercellular and Intracellular Signaling Pathways Mediating Ionizing Radiation‐Induced Bystander Effects.” Journal of Radiation Research 48, no. 2: 87–95. 17327686 10.1269/jrr.06084

[em70031-bib-0068] Hein, A. L. , M. M. Ouellette , and Y. Yan . 2014. “Radiation‐Induced Signaling Pathways That Promote Cancer Cell Survival (Review).” International Journal of Oncology 45, no. 5: 1813–1819. 25174607 10.3892/ijo.2014.2614 PMC4203326

[em70031-bib-0069] Hladik, D. , and S. Tapio . 2016. “Effects of Ionizing Radiation on the Mammalian Brain.” Mutation Research, Reviews in Mutation Research 770, no. Pt B: 219–230. 27919332 10.1016/j.mrrev.2016.08.003

[em70031-bib-0070] Hsu, T. , H. H. Nguyen‐Tran , and M. Trojanowska . 2019. “Active Roles of Dysfunctional Vascular Endothelium in Fibrosis and Cancer.” Journal of Biomedical Science 26, no. 1: 86. 31656195 10.1186/s12929-019-0580-3 PMC6816223

[em70031-bib-0071] Huff, J. L. , I. Plante , S. R. Blattnig , et al. 2022. “Cardiovascular Disease Risk Modeling for Astronauts: Making the Leap From Earth to Space.” Frontiers in Cardiovascular Medicine 9: 873597. 35665268 10.3389/fcvm.2022.873597 PMC9161032

[em70031-bib-0072] Huff, J. L. , F. Poignant , S. Rahmanian , et al. 2023. “Galactic Cosmic Ray Simulation at the NASA Space Radiation Laboratory ‐ Progress, Challenges and Recommendations on Mixed‐Field Effects.” Life Sciences in Space Research 36: 90–104. 36682835 10.1016/j.lssr.2022.09.001

[em70031-bib-0073] Hughson, R. L. , A. Helm , and M. Durante . 2018. “Heart in Space: Effect of the Extraterrestrial Environment on the Cardiovascular System.” Nature Reviews. Cardiology 15, no. 3: 167–180. 29053152 10.1038/nrcardio.2017.157

[em70031-bib-0074] Hunsberger, H. C. , P. D. Pinky , W. Smith , V. Suppiramaniam , and M. N. Reed . 2019. “The Role of APOE4 in Alzheimer's Disease: Strategies for Future Therapeutic Interventions.” Neuronal Signaling 3, no. 2: NS20180203. 32269835 10.1042/NS20180203 PMC7104324

[em70031-bib-0007] ICRP , F. A. Stewart , A. V. Akleyev , et al. 2012. “ICRP Publication 118: ICRP Statement on Tissue Reactions and Early and Late Effects of Radiation in Normal Tissues and Organs‐Threshold Doses for Tissue Reactions in a Radiation Protection Context.” Annals of the ICRP 41, no. 1–2: 1–322. 10.1016/j.icrp.2012.02.001 22925378

[em70031-bib-0075] Ivanov, V. K. , M. A. Maksioutov , S. Y. Chekin , et al. 2006. “The Risk of Radiation‐Induced Cerebrovascular Disease in Chernobyl Emergency Workers.” Health Physics 90, no. 3: 199–207. 16505616 10.1097/01.HP.0000175835.31663.ea

[em70031-bib-0076] Jilka, R. L. , B. Noble , and R. S. Weinstein . 2013. “Osteocyte Apoptosis.” Bone 54, no. 2: 264–271. 23238124 10.1016/j.bone.2012.11.038 PMC3624050

[em70031-bib-0077] Johnson, I. R. D. , C. T. Nguyen , P. Wise , and D. Grimm . 2020. “Implications of Altered Endosome and Lysosome Biology in Space Environments.” International Journal of Molecular Sciences 21, no. 21: 8205. 33147843 10.3390/ijms21218205 PMC7663135

[em70031-bib-0078] Jones, C. W. , E. G. Overbey , J. Lacombe , et al. 2024. “Molecular and Physiological Changes in the SpaceX Inspiration4 Civilian Crew.” Nature 632, no. 8027: 1155–1164. 38862026 10.1038/s41586-024-07648-x PMC11357997

[em70031-bib-0079] Jones, J. A. , M. McCarten , K. Manuel , et al. 2006. “Understanding Cataract Risk in Aerospace Flight Crew and Review of Mechanisms of Cataract Formation.” https://ntrs.nasa.gov/citations/20060051793.

[em70031-bib-0080] Jovanovic, S. V. , and M. G. Simic . 1986. “One‐Electron Redox Potentials of Purines and Pyrimidines.” Journal of Physical Chemistry 90: 974–978.

[em70031-bib-0081] Kashcheev, V. V. , S. Y. Chekin , S. V. Karpenko , et al. 2017. “Radiation Risk of Cardiovascular Diseases in the Cohort of Russian Emergency Workers of the Chernobyl Accident.” Health Physics 113, no. 1: 23–29. 28542008 10.1097/HP.0000000000000670

[em70031-bib-0082] Kennedy, E. M. , D. R. Powell , Z. Li , et al. 2018. “Galactic Cosmic Radiation Induces Persistent Epigenome Alterations Relevant to Human Lung Cancer.” Scientific Reports 8, no. 1: 6709. 29712937 10.1038/s41598-018-24755-8 PMC5928241

[em70031-bib-0083] Khalid, Y. , M. Fradley , N. Dasu , K. Dasu , A. Shah , and A. Levine . 2020. “Gender Disparity in Cardiovascular Mortality Following Radiation Therapy for Hodgkin's Lymphoma: A Systematic Review.” Cardio‐Oncology 6: 12. 32774890 10.1186/s40959-020-00067-7 PMC7405444

[em70031-bib-0084] Khan, M. G. M. , and Y. Wang . 2022. “Advances in the Current Understanding of How Low‐Dose Radiation Affects the Cell Cycle.” Cells 11, no. 3: 356. 35159169 10.3390/cells11030356 PMC8834401

[em70031-bib-0085] Klokov, D. , K. Applegate , C. Badie , et al. 2022. “International Expert Group Collaboration for Developing an Adverse Outcome Pathway for Radiation Induced Leukemia.” International Journal of Radiation Biology 98, no. 12: 1802–1815. 36040845 10.1080/09553002.2022.2117873

[em70031-bib-0086] Komori, T. 2013. “Functions of the Osteocyte Network in the Regulation of Bone Mass.” Cell and Tissue Research 352, no. 2: 191–198. 23329124 10.1007/s00441-012-1546-x PMC3637644

[em70031-bib-0087] Kozbenko, T. , N. Adam , V. S. Grybas , et al. 2024. “AOP Report: Development of an Adverse Outcome Pathway for Deposition of Energy Leading to Abnormal Vascular Remodeling.” Environmental and Molecular Mutagenesis 65, no. S3: 4–30. 10.1002/em.22636 39440813

[em70031-bib-0088] Kozbenko, T. , N. Adam , V. Lai , et al. 2022. “Deploying Elements of Scoping Review Methods for Adverse Outcome Pathway Development: A Space Travel Case Example.” International Journal of Radiation Biology 98, no. 12: 1777–1788. 35939057 10.1080/09553002.2022.2110306

[em70031-bib-0089] Krüger‐Genge, A. , A. Blocki , R. P. Franke , and F. Jung . 2019. “Vascular Endothelial Cell Biology: An Update.” International Journal of Molecular Sciences 20, no. 18: 4411. 31500313 10.3390/ijms20184411 PMC6769656

[em70031-bib-0090] Krukowski, K. , K. Grue , E. S. Frias , et al. 2018. “Female Mice Are Protected From Space Radiation‐Induced Maladaptive Responses.” Brain, Behavior, and Immunity 74: 106–120. 30107198 10.1016/j.bbi.2018.08.008 PMC8715721

[em70031-bib-0091] Krull, K. R. , K. K. Hardy , L. S. Kahalley , I. Schuitema , and S. R. Kesler . 2018. “Neurocognitive Outcomes and Interventions in Long‐Term Survivors of Childhood Cancer.” Journal of Clinical Oncology: Official Journal of the American Society of Clinical Oncology 36, no. 21: 2181–2189. 29874137 10.1200/JCO.2017.76.4696 PMC6553837

[em70031-bib-0092] Lang, T. , J. J. W. A. Van Loon , S. Bloomfield , et al. 2017. “Towards Human Exploration of Space: The THESEUS Review Series on Muscle and Bone Research Priorities.” NPJ Microgravity 3: 8. 28649630 10.1038/s41526-017-0013-0 PMC5445590

[em70031-bib-0093] Lee, S. M. C. , L. C. Ribeiro , D. S. Martin , et al. 2020. “Arterial Structure and Function During and After Long‐Duration Spaceflight.” Journal of Applied Physiology 129, no. 1: 108–123. 32525433 10.1152/japplphysiol.00550.2019

[em70031-bib-0094] Lehtinen, M. K. , and A. Bonni . 2006. “Modeling Oxidative Stress in the Central Nervous System.” Current Molecular Medicine 6, no. 8: 871–881. 17168738 10.2174/156652406779010786

[em70031-bib-0095] Lestaevel, P. , S. Grison , G. Favé , et al. 2016. “Assessment of the Central Effects of Natural Uranium via Behavioural Performances and the Cerebrospinal Fluid Metabolome.” Neural Plasticity 2016: 9740353. 27247806 10.1155/2016/9740353 PMC4877492

[em70031-bib-0096] Li, R. , W. Yang , X. Hu , et al. 2020. “Effect of Autophagy on Irradiation‐Induced Damage in Osteoblast‐Like MC3T3‐E1 Cells.” Molecular Medicine Reports 22, no. 4: 3473–3481. 32945432 10.3892/mmr.2020.11425 PMC7453677

[em70031-bib-0097] Little, M. P. , T. V. Azizova , D. B. Richardson , et al. 2023. “Ionising Radiation and Cardiovascular Disease: Systematic Review and Meta‐Analysis.” British Medical Journal 380: e072924. 36889791 10.1136/bmj-2022-072924 PMC10535030

[em70031-bib-0098] Little, M. P. , M. Boerma , M. O. Bernier , et al. 2024. “Effects of Confounding and Effect‐Modifying Lifestyle, Environmental and Medical Factors on Risk of Radiation‐Associated Cardiovascular Disease.” BMC Public Health 24, no. 1: 1601. 10.1186/s12889-024-18701-9. 38879521 PMC11179258

[em70031-bib-0099] Liu, H. , A. J. Smith , M. C. Lott , et al. 2013. “Sulforaphane Can Protect Lens Cells Against Oxidative Stress: Implications for Cataract Prevention.” Investigative Ophthalmology & Visual Science 54, no. 8: 5236–5248. 10.1167/iovs.13-11664. 23812493

[em70031-bib-0100] Luiking, Y. C. , M. P. Engelen , and N. E. Deutz . 2010. “Regulation of Nitric Oxide Production in Health and Disease.” Current Opinion in Clinical Nutrition and Metabolic Care 13, no. 1: 97–104. 19841582 10.1097/MCO.0b013e328332f99d PMC2953417

[em70031-bib-0101] Mahley, R. W. 2016. “Apolipoprotein E: From Cardiovascular Disease to Neurodegenerative Disorders.” Journal of Molecular Medicine 94, no. 7: 739–746. 27277824 10.1007/s00109-016-1427-y PMC4921111

[em70031-bib-0102] Man, J. , T. Graham , G. Squires‐Donelly , and A. L. Laslett . 2022. “The Effects of Microgravity on Bone Structure and Function.” NPJ Microgravity 8, no. 1: 9. 35383182 10.1038/s41526-022-00194-8 PMC8983659

[em70031-bib-0103] Manda, K. , K. Anzai , S. Kumari , and A. L. Bhatia . 2007. “Melatonin Attenuates Radiation‐Induced Learning Deficit and Brain Oxidative Stress in Mice.” Acta Neurobiologiae Experimentalis 67, no. 1: 63–70. 17474322 10.55782/ane-2007-1633

[em70031-bib-0104] Markkanen, E. 2017. “Not Breathing Is Not an Option: How to Deal With Oxidative DNA Damage.” DNA Repair 59: 82–105. 28963982 10.1016/j.dnarep.2017.09.007

[em70031-bib-0105] Mason, C. E. , J. Green , K. I. Adamopoulos , et al. 2024. “A Second Space Age Spanning Omics, Platforms and Medicine Across Orbits.” Nature 632, no. 8027: 995–1008. 38862027 10.1038/s41586-024-07586-8 PMC12366838

[em70031-bib-0106] Mauvais‐Jarvis, F. , A. P. Arnold , and K. Reue . 2017. “A Guide for the Design of Pre‐Clinical Studies on Sex Differences in Metabolism.” Cell Metabolism 25, no. 6: 1216–1230. 28591630 10.1016/j.cmet.2017.04.033 PMC5516948

[em70031-bib-0107] Mavragani, I. V. , D. A. Laskaratou , B. Frey , et al. 2015. “Key Mechanisms Involved in Ionizing Radiation‐Induced Systemic Effects. A Current Review.” Toxicology Research 5, no. 1: 12–33. 30090323 10.1039/c5tx00222b PMC6061884

[em70031-bib-0108] Mavragani, I. V. , Z. Nikitaki , S. A. Kalospyros , and A. G. Georgakilas . 2019. “Ionizing Radiation and Complex DNA Damage: From Prediction to Detection Challenges and Biological Significance.” Cancers 11, no. 11: 1789. 31739493 10.3390/cancers11111789 PMC6895987

[em70031-bib-0109] McCarron, R. A. , S. G. R. Barnard , G. Babini , et al. 2022. “Radiation‐Induced Lens Opacity and Cataractogenesis: A Lifetime Study Using Mice of Varying Genetic Backgrounds.” Radiation Research 197, no. 1: 57–66. 33984859 10.1667/RADE-20-00266.1

[em70031-bib-0110] Mehare, A. , S. Chakole , and B. Wandile . 2024. “Navigating the Unknown: A Comprehensive Review of Spaceflight‐Associated Neuro‐Ocular Syndrome.” Cureus 16, no. 2: e53380. 38435236 10.7759/cureus.53380 PMC10907968

[em70031-bib-0111] Memme, J. M. , M. Slavin , N. Moradi , and D. A. Hood . 2021. “Mitochondrial Bioenergetics and Turnover During Chronic Muscle Disuse.” International Journal of Molecular Sciences 22, no. 10: 5179. 34068411 10.3390/ijms22105179 PMC8153634

[em70031-bib-0112] Minamoto, A. , H. Taniguchi , N. Yoshitani , et al. 2004. “Cataract in Atomic Bomb Survivors.” International Journal of Radiation Biology 80, no. 5: 339–345. 15223766 10.1080/09553000410001680332

[em70031-bib-0113] Mitchell, A. , D. Pimenta , J. Gill , H. Ahmad , and R. Bogle . 2019. “Cardiovascular Effects of Space Radiation: Implications for Future Human Deep Space Exploration.” European Journal of Preventive Cardiology 26, no. 16: 1707–1714. 30776915 10.1177/2047487319831497

[em70031-bib-0114] Mitchell, B. D. , and L. M. Yerges‐Armstrong . 2011. “The Genetics of Bone Loss: Challenges and Prospects.” Journal of Clinical Endocrinology and Metabolism 96, no. 5: 1258–1268. 21346070 10.1210/jc.2010-2865 PMC3085199

[em70031-bib-0115] Mittal, M. , M. R. Siddiqui , K. Tran , S. P. Reddy , and A. B. Malik . 2014. “Reactive Oxygen Species in Inflammation and Tissue Injury.” Antioxidants & Redox Signaling 20, no. 7: 1126–1167. 23991888 10.1089/ars.2012.5149 PMC3929010

[em70031-bib-0116] Monje, M. L. , and T. Palmer . 2003. “Radiation Injury and Neurogenesis.” Current Opinion in Neurology 16, no. 2: 129–134. 12644738 10.1097/01.wco.0000063772.81810.b7

[em70031-bib-0117] Mozaffarian, D. , P. W. Wilson , and W. B. Kannel . 2008. “Beyond Established and Novel Risk Factors: Lifestyle Risk Factors for Cardiovascular Disease.” Circulation 117, no. 23: 3031–3038. 18541753 10.1161/CIRCULATIONAHA.107.738732

[em70031-bib-0118] Nagane, M. , H. Yasui , P. Kuppusamy , T. Yamashita , and O. Inanami . 2021. “DNA Damage Response in Vascular Endothelial Senescence: Implication for Radiation‐Induced Cardiovascular Diseases.” Journal of Radiation Research 62, no. 4: 564–573. 33912932 10.1093/jrr/rrab032 PMC8273807

[em70031-bib-0119] NCRP . 2020. “Approaches for Integrating Information From Radiation Biology and Epidemiology to Enhance Low‐Dose Health Risk Assessment.” NCRP Report No. 186, National Council on Radiation Protection and Measurements. 2020.

[em70031-bib-0120] Nelson, C. P. , A. Goel , A. S. Butterworth , et al. 2017. “Association Analyses Based on False Discovery Rate Implicate New Loci for Coronary Artery Disease.” Nature Genetics 49, no. 9: 1385–1391. 28714975 10.1038/ng.3913

[em70031-bib-0121] Nikjoo, H. , D. Emfietzoglou , T. Liamsuwan , R. Taleei , D. Liljequist , and S. Uehara . 2016. “Radiation Track, DNA Damage and Response‐A Review.” Reports on Progress in Physics 79, no. 11: 116601. 27652826 10.1088/0034-4885/79/11/116601

[em70031-bib-0122] Nikpay, M. , A. Goel , H. H. Won , et al. 2015. “A Comprehensive 1,000 Genomes‐Based Genome‐Wide Association Meta‐Analysis of Coronary Artery Disease.” Nature Genetics 47, no. 10: 1121–1130. 26343387 10.1038/ng.3396 PMC4589895

[em70031-bib-0123] Norbury, J. W. , W. Schimmerling , T. C. Slaba , et al. 2016. “Galactic Cosmic Ray Simulation at the NASA Space Radiation Laboratory.” Life Sciences in Space Research 8: 38–51. 26948012 10.1016/j.lssr.2016.02.001 PMC5771487

[em70031-bib-0124] North, B. J. , and D. A. Sinclair . 2012. “The Intersection Between Aging and Cardiovascular Disease.” Circulation Research 110, no. 8: 1097–1108. 22499900 10.1161/CIRCRESAHA.111.246876 PMC3366686

[em70031-bib-0125] OECD . 2018. “Users' Handbook Supplement to the Guidance Document for Developing and Assessing Adverse Outcome Pathways, OECD Series on Adverse Outcome Pathways, Organisation for Economic Cooperation and Development, Paris, France.”

[em70031-bib-0126] Oeffinger, K. C. , A. C. Mertens , C. A. Sklar , et al. 2006. “Chronic Health Conditions in Adult Survivors of Childhood Cancer.” New England Journal of Medicine 355, no. 15: 1572–1582. 17035650 10.1056/NEJMsa060185

[em70031-bib-0127] Olivieri, F. , R. Recchioni , F. Marcheselli , et al. 2013. “Cellular Senescence in Cardiovascular Diseases: Potential Age‐Related Mechanisms and Implications for Treatment.” Current Pharmaceutical Design 19, no. 9: 1710–1719. 23061728

[em70031-bib-0128] Ostman, B. , K. Michaëlsson , J. Helmersson , et al. 2009. “Oxidative Stress and Bone Mineral Density in Elderly Men: Antioxidant Activity of Alpha‐Tocopherol.” Free Radical Biology & Medicine 47, no. 5: 668–673. 19500667 10.1016/j.freeradbiomed.2009.05.031

[em70031-bib-0129] Ott, M. , V. Gogvadze , S. Orrenius , and B. Zhivotovsky . 2007. “Mitochondria, Oxidative Stress and Cell Death.” Apoptosis 12, no. 5: 913–922. 17453160 10.1007/s10495-007-0756-2

[em70031-bib-0130] Overbey, E. G. , J. Kim , B. T. Tierney , et al. 2024. “The Space Omics and Medical Atlas (SOMA) and International Astronaut Biobank.” Nature 632, no. 8027: 1145–1154. 38862028 10.1038/s41586-024-07639-y PMC11357981

[em70031-bib-0131] Ozasa, K. , Y. Shimizu , A. Suyama , et al. 2012. “Studies of the Mortality of Atomic Bomb Survivors, Report 14, 1950‐2003: An Overview of Cancer and Noncancer Diseases.” Radiation Research 177, no. 3: 229–243. 22171960 10.1667/rr2629.1

[em70031-bib-0132] Pacheco, R. , and H. Stock . 2013. “Effects of Radiation on Bone.” Current Osteoporosis Reports 11, no. 4: 299–304. 24057133 10.1007/s11914-013-0174-z

[em70031-bib-0133] Pasqual, E. , F. Boussin , D. Bazyka , et al. 2021. “Cognitive Effects of Low Dose of Ionizing Radiation – Lessons Learned and Research Gaps From Epidemiological and Biological Studies.” Environment International 147: 106295. 33341586 10.1016/j.envint.2020.106295

[em70031-bib-0134] Patel, Z. S. , T. J. Brunstetter , W. J. Tarver , et al. 2020. “Red Risks for a Journey to the Red Planet: The Highest Priority Human Health Risks for a Mission to Mars.” NPJ Microgravity 6, no. 1: 33. 33298950 10.1038/s41526-020-00124-6 PMC7645687

[em70031-bib-0135] Ping, Z. , Y. Peng , H. Lang , et al. 2020. “Oxidative Stress in Radiation‐Induced Cardiotoxicity.” Oxidative Medicine and Cellular Longevity 2020: 3579143. 32190171 10.1155/2020/3579143 PMC7071808

[em70031-bib-0136] Pitolli, C. , Y. Wang , M. Mancini , Y. Shi , G. Melino , and I. Amelio . 2019. “Do Mutations Turn p53 Into an Oncogene?” International Journal of Molecular Sciences 20, no. 24: 6241. 31835684 10.3390/ijms20246241 PMC6940991

[em70031-bib-0137] Preston, D. L. , Y. Shimizu , D. A. Pierce , A. Suyama , and K. Mabuchi . 2003. “Studies of Mortality of Atomic Bomb Survivors. Report 13: Solid Cancer and Noncancer Disease Mortality: 1950‐1997.” Radiation Research 160, no. 4: 381–407. 12968934 10.1667/rr3049

[em70031-bib-0138] Prise, K. M. , C. H. Pullar , and B. D. Michael . 1999. “A Study of Endonuclease III‐Sensitive Sites in Irradiated DNA: Detection of Alpha‐Particle‐Induced Oxidative Damage.” Carcinogenesis 20, no. 5: 905–909. 10334210 10.1093/carcin/20.5.905

[em70031-bib-0139] Rachal Pugh, C. , M. Fleshner , L. R. Watkins , S. F. Maier , and J. W. Rudy . 2001. “The Immune System and Memory Consolidation: A Role for the Cytokine IL‐1beta.” Neuroscience and Biobehavioral Reviews 25, no. 1: 29–41. 11166076 10.1016/s0149-7634(00)00048-8

[em70031-bib-0140] Ramalingam, M. , and S. J. Kim . 2012. “Reactive Oxygen/Nitrogen Species and Their Functional Correlations in Neurodegenerative Diseases.” Journal of Neural Transmission 119, no. 8: 891–910. 22212484 10.1007/s00702-011-0758-7

[em70031-bib-0141] Rana, T. , M. A. Schultz , M. L. Freeman , and S. Biswas . 2012. “Loss of Nrf2 Accelerates Ionizing Radiation‐Induced Bone Loss by Upregulating RANKL.” Free Radical Biology & Medicine 53, no. 12: 2298–2307. 23085426 10.1016/j.freeradbiomed.2012.10.536 PMC3762920

[em70031-bib-0142] Ranganathan, P. 2009. “Genetics of Bone Loss in Rheumatoid Arthritis – Role of Vitamin D Receptor Polymorphisms.” Rheumatology 48, no. 4: 342–346. 19151030 10.1093/rheumatology/ken473 PMC2722799

[em70031-bib-0143] Rao, S. B. 2016. “Biological Bases for the Revision of Dose Limits to the Eye Lens.” Journal of Medical Physics 41, no. 4: 211–213. 28144111 10.4103/0971-6203.195183 PMC5228042

[em70031-bib-0144] Richardson, R. B. 2022. “The Role of Oxygen and the Goldilocks Range in the Development of Cataracts Induced by Space Radiation in US Astronauts.” Experimental Eye Research 223: 109192. 35917999 10.1016/j.exer.2022.109192

[em70031-bib-0145] Richardson, R. B. , E. A. Ainsbury , C. R. Prescott , and F. J. Lovicu . 2020. “Etiology of Posterior Subcapsular Cataracts Based on a Review of Risk Factors Including Aging, Diabetes, and Ionizing Radiation.” International Journal of Radiation Biology 96, no. 11: 1339–1361. 32897800 10.1080/09553002.2020.1812759

[em70031-bib-0146] Russell, N. S. , S. Hoving , S. Heeneman , et al. 2009. “Novel Insights Into Pathological Changes in Muscular Arteries of Radiotherapy Patients.” Radiotherapy and Oncology 92, no. 3: 477–483. 19541382 10.1016/j.radonc.2009.05.021

[em70031-bib-0147] Rydberg, B. 1996. “Clusters of DNA Damage Induced by Ionizing Radiation: Formation of Short DNA Fragments. II. Experimental Detection.” Radiation Research 145, no. 2: 200–209. 8606930

[em70031-bib-0148] Sandhu, S. , M. Keyworth , S. Karimi‐Jashni , et al. 2024. “AOP Report: Development of an Adverse Outcome Pathway for Deposition of Energy Leading to Bone Loss.” Environmental and Molecular Mutagenesis 65, no. S3: 85–111. 39387375 10.1002/em.22631

[em70031-bib-0149] Sasi, S. P. , D. Park , S. Muralidharan , et al. 2015. “Particle Radiation‐Induced Nontargeted Effects in Bone‐Marrow‐Derived Endothelial Progenitor Cells.” Stem Cells International 2015: 496512. 26074973 10.1155/2015/496512 PMC4436457

[em70031-bib-0150] Schaue, D. , E. D. Micewicz , J. A. Ratikan , M. W. Xie , G. Cheng , and W. H. McBride . 2015. “Radiation and Inflammation.” Seminars in Radiation Oncology 25, no. 1: 4–10. 25481260 10.1016/j.semradonc.2014.07.007 PMC4378687

[em70031-bib-0151] Schiffrin, E. L. 2008. “Oxidative Stress, Nitric Oxide Synthase, and Superoxide Dismutase: A Matter of Imbalance Underlies Endothelial Dysfunction in the Human Coronary Circulation.” Hypertension 51, no. 1: 31–32. 18071058 10.1161/HYPERTENSIONAHA.107.103226

[em70031-bib-0152] Schoenfeld, M. P. , R. R. Ansari , A. Nakao , and D. Wink . 2012. “A Hypothesis on Biological Protection From Space Radiation Through the Use of New Therapeutic Gases as Medical Counter Measures.” Medical Gas Research 2: 8. 22475015 10.1186/2045-9912-2-8 PMC3348081

[em70031-bib-0153] Senoner, T. , and W. Dichtl . 2019. “Oxidative Stress in Cardiovascular Diseases: Still a Therapeutic Target?” Nutrients 11, no. 9: 2090. 31487802 10.3390/nu11092090 PMC6769522

[em70031-bib-0154] Sherman, S. , Z. Said , B. Sadi , et al. 2023. Adverse Outcome Pathway on Deposition of Energy Leading to Lung Cancer, OECD Series on Adverse Outcome Pathways, No. 32. OECD Publishing. 10.1787/a8f262c2-en.

[em70031-bib-0155] Shimizu, Y. , K. Kodama , N. Nishi , et al. 2010. “Radiation Exposure and Circulatory Disease Risk: Hiroshima and Nagasaki Atomic Bomb Survivor Data, 1950–2003.” British Medical Journal 340: b5349. 20075151 10.1136/bmj.b5349 PMC2806940

[em70031-bib-0156] Sibonga, J. , T. Matsumoto , J. Jones , et al. 2019. “Resistive Exercise in Astronauts on Prolonged Spaceflights Provides Partial Protection Against Spaceflight‐Induced Bone Loss.” Bone 128: 112037. 31400472 10.1016/j.bone.2019.07.013

[em70031-bib-0157] Sleiman, A. , K. B. Miller , D. Flores , et al. 2024. “AOP Report: Development of an Adverse Outcome Pathway for Deposition of Energy Leading to Learning and Memory Impairment.” Environmental and Molecular Mutagenesis 65, no. S3: 57–84. 10.1002/em.22622 39228295

[em70031-bib-0158] Slezak, J. , B. Kura , P. Babal , et al. 2017. “Potential Markers and Metabolic Processes Involved in the Mechanism of Radiation‐Induced Heart Injury.” Canadian Journal of Physiology and Pharmacology 95, no. 10: 1190–1203. 28750189 10.1139/cjpp-2017-0121

[em70031-bib-0159] Smith, J. K. 2020. “Microgravity, Bone Homeostasis, and Insulin‐Like Growth Factor‐1.” Applied Sciences 10, no. 13: 4433.

[em70031-bib-0160] Soloviev, A. I. , and I. V. Kizub . 2019. “Mechanisms of Vascular Dysfunction Evoked by Ionizing Radiation and Possible Targets for Its Pharmacological Correction.” Biochemical Pharmacology 159: 121–139. 30508525 10.1016/j.bcp.2018.11.019

[em70031-bib-0161] Song, T. T. , R. S. Cai , R. Hu , Y. S. Xu , B. N. Qi , and Y. A. Xiong . 2021. “The Important Role of TFEB in Autophagy‐Lysosomal Pathway and Autophagy‐Related Diseases: A Systematic Review.” European Review for Medical and Pharmacological Sciences 25, no. 3: 1641–1649. 33629334 10.26355/eurrev_202102_24875

[em70031-bib-0162] Stavnichuk, M. , N. Mikolajewicz , T. Corlett , M. Morris , and S. V. Komarova . 2020. “A Systematic Review and Meta‐Analysis of Bone Loss in Space Travelers.” NPJ Microgravity 6: 13. 32411816 10.1038/s41526-020-0103-2 PMC7200725

[em70031-bib-0163] Sutherland, B. M. , P. V. Bennett , O. Sidorkina , and J. Laval . 2000. “Clustered DNA Damages Induced in Isolated DNA and in Human Cells by Low Doses of Ionizing Radiation.” Proceedings of the National Academy of Sciences of The United States of America 97, no. 1: 103–108. 10618378 10.1073/pnas.97.1.103 PMC26623

[em70031-bib-0164] Sylvester, C. B. , J. I. Abe , Z. S. Patel , and K. J. Grande‐Allen . 2018. “Radiation‐Induced Cardiovascular Disease: Mechanisms and Importance of Linear Energy Transfer.” Frontiers in Cardiovascular Medicine 5: 5. 29445728 10.3389/fcvm.2018.00005 PMC5797745

[em70031-bib-0165] Tahimic, C. G. T. , and R. K. Globus . 2017. “Redox Signaling and Its Impact on Skeletal and Vascular Responses to Spaceflight.” International Journal of Molecular Sciences 18, no. 10: 2153. 29035346 10.3390/ijms18102153 PMC5666834

[em70031-bib-0166] Takahashi, I. , Y. Shimizu , E. J. Grant , J. Cologne , K. Ozasa , and K. Kodama . 2017. “Heart Disease Mortality in the Life Span Study, 1950‐2008.” Radiation Research 187, no. 3: 319–332. 28170314 10.1667/RR14347.1

[em70031-bib-0167] Tang, F. R. , W. K. Loke , and B. C. Khoo . 2017. “Postnatal Irradiation‐Induced Hippocampal Neuropathology, Cognitive Impairment and Aging.” Brain & Development 39, no. 4: 277–293. 27876394 10.1016/j.braindev.2016.11.001

[em70031-bib-0168] Tangvarasittichai, O. , and S. Tangvarasittichai . 2018. “Oxidative Stress, Ocular Disease and Diabetes Retinopathy.” Current Pharmaceutical Design 24, no. 40: 4726–4741. 30644339 10.2174/1381612825666190115121531

[em70031-bib-0169] Taormina, D. P. , S. Rozenblatt , L. T. Guey , et al. 2008. “The Chornobyl Accident and Cognitive Functioning: A Follow‐Up Study of Infant Evacuees at Age 19 Years.” Psychological Medicine 38, no. 4: 489–497. 18177528 10.1017/S0033291707002462

[em70031-bib-0170] Task Group on Radiation Protection in Space , ICRP Committee 2 , G. Dietze , et al. 2013. “Assessment of Radiation Exposure of Astronauts in Space. ICRP Publication 123.” Annals of the ICRP 42, no. 4: 1–339. 10.1016/j.icrp.2013.05.004 23958389

[em70031-bib-0171] Thiagarajan, R. , and R. Manikandan . 2013. “Antioxidants and Cataract.” Free Radical Research 47, no. 5: 337–345. 23438873 10.3109/10715762.2013.777155

[em70031-bib-0172] Timm, S. , Y. Lorat , B. Jakob , G. Taucher‐Scholz , and C. E. Rübe . 2018. “Clustered DNA Damage Concentrated in Particle Trajectories Causes Persistent Large‐Scale Rearrangements in Chromatin Architecture.” Radiotherapy and Oncology: Journal of the European Society for Therapeutic Radiology and Oncology 129, no. 3: 600–610. 30049456 10.1016/j.radonc.2018.07.003

[em70031-bib-0173] Townsend, L. W. 2005. “Implications of the Space Radiation Environment for Human Exploration in Deep Space.” Radiation Protection Dosimetry 115, no. 1–4: 44–50. 16381680 10.1093/rpd/nci141

[em70031-bib-0174] Turker, M. S. , D. Grygoryev , M. Lasarev , et al. 2017. “Simulated Space Radiation‐Induced Mutants in the Mouse Kidney Display Widespread Genomic Change.” PLoS One 12, no. 7: e0180412. 28683078 10.1371/journal.pone.0180412 PMC5500326

[em70031-bib-0175] Turner, C. H. 2002. “Biomechanics of Bone: Determinants of Skeletal Fragility and Bone Quality.” Osteoporosis International 13, no. 2: 97–104. 11905527 10.1007/s001980200000

[em70031-bib-0176] Turner, N. D. , L. A. Braby , J. Ford , and J. R. Lupton . 2002. “Opportunities for Nutritional Amelioration of Radiation‐Induced Cellular Damage.” Nutrition 18, no. 10: 904–912. 10.1016/s0899-9007(02)00945-0. 12361786

[em70031-bib-0177] Venkatesulu, B. P. , L. S. Mahadevan , M. L. Aliru , et al. 2018. “Radiation‐Induced Endothelial Vascular Injury: A Review of Possible Mechanisms.” Journal of the American College of Cardiology 3, no. 4: 563–572. 10.1016/j.jacbts.2018.01.014 PMC6115704 30175280

[em70031-bib-0178] Vernice, N. A. , C. Meydan , E. Afshinnekoo , and C. E. Mason . 2020. “Long‐Term Spaceflight and the Cardiovascular System.” Precision Clinical Medicine 3, no. 4: 284–291. 33391848 10.1093/pcmedi/pbaa022 PMC7757439

[em70031-bib-0179] Vieira Dias, J. , C. Gloaguen , D. Kereselidze , L. Manens , K. Tack , and T. G. Ebrahimian . 2018. “Gamma Low‐Dose‐Rate Ionizing Radiation Stimulates Adaptive Functional and Molecular Response in Human Aortic Endothelial Cells in a Threshold‐, Dose‐, and Dose Rate‐Dependent Manner.” Dose‐Response: A Publication of International Hormesis Society 16, no. 1: 1559325818755238. 29531508 10.1177/1559325818755238 PMC5843109

[em70031-bib-0180] Villeneuve, D. L. , D. Crump , N. Garcia‐Reyero , et al. 2014. “Adverse Outcome Pathway Development II: Best Practices.” Toxicological sciences: an official journal of the Society of Toxicology 142, no. 2: 321–330. 25466379 10.1093/toxsci/kfu200 PMC4318924

[em70031-bib-0181] Vitale, C. , M. E. Mendelsohn , and G. M. Rosano . 2009. “Gender Differences in the Cardiovascular Effect of Sex Hormones.” Nature Reviews Cardiology 6, no. 8: 532–542. 19564884 10.1038/nrcardio.2009.105

[em70031-bib-0182] Vuong, N. V. , S. Khilji , A. Williams , et al. 2025. “Integration of Multi‐Omics and Benchmark Dose Modeling to Support Adverse Outcome Pathways.” International Journal of Radiation Biology 101, no. 3: 240–253. 39746153 10.1080/09553002.2024.2442694

[em70031-bib-0183] Wang, H. , J. Wei , Q. Zheng , et al. 2019. “Radiation‐Induced Heart Disease: A Review of Classification, Mechanism and Prevention.” International Journal of Biological Sciences 15, no. 10: 2128–2138. 31592122 10.7150/ijbs.35460 PMC6775290

[em70031-bib-0184] Wang, S. , Z. Deng , Y. Ma , et al. 2020. “The Role of Autophagy and Mitophagy in Bone Metabolic Disorders.” International Journal of Biological Sciences 16, no. 14: 2675–2691. 32792864 10.7150/ijbs.46627 PMC7415419

[em70031-bib-0185] Warden, D. , and Y. Bayazitoglu . 2019. “New Comparative Metric for Evaluating Spacecraft Radiation Shielding.” Journal of Spacecraft and Rockets 56: 1024–1038.

[em70031-bib-0186] Willey, J. S. , S. A. Lloyd , G. A. Nelson , and T. A. Bateman . 2011. “Ionizing Radiation and Bone Loss: Space Exploration and Clinical Therapy Applications.” Clinical Reviews in Bone and Mineral Metabolism 9, no. 1: 54–62. 22826690 10.1007/s12018-011-9092-8 PMC3401480

[em70031-bib-0187] Willey, J. S. , S. A. J. Lloyd , and T. A. Bateman . 2013. “Radiation Therapy‐Induced Osteoporosis.” In Primer on the Metabolic Bone Diseases and Disorders of Mineral Metabolism, 8th ed., 728–733. John Wiley & Sons, Inc: ASBMR.

[em70031-bib-0188] Williams, H. J. , and A. M. Davies . 2006. “The Effect of X‐Rays on Bone: A Pictorial Review.” European Radiology 16, no. 3: 619–633. 16237551 10.1007/s00330-005-0010-7

[em70031-bib-0189] Wissing, M. D. 2015. “Chemotherapy‐ and Irradiation‐Induced Bone Loss in Adults With Solid Tumors.” Current Osteoporosis Reports 13, no. 3: 140–145. 25712619 10.1007/s11914-015-0266-z PMC4417126

[em70031-bib-0190] Worgul, B. V. , Y. I. Kundiyev , N. M. Sergiyenko , et al. 2007. “Cataracts Among Chernobyl Clean‐Up Workers: Implications Regarding Permissible Eye Exposures.” Radiation Research 167, no. 2: 233–243. 17390731 10.1667/rr0298.1

[em70031-bib-0191] Wright, L. E. 2018. “Primer on the Metabolic Bone Diseases and Disorders of Mineral Metabolism.” In Radiotherapy‐Induced Osteoporosis, 9th ed., 788–792. John Wiley & Sons, Inc: ASBMR.

[em70031-bib-0192] Xiong, J. , and C. A. O'Brien . 2012. “Osteocyte RANKL: New Insights Into the Control of Bone Remodeling.” Journal of Bone and Mineral Research: the Official Journal of the American Society for Bone and Mineral Research 27, no. 3: 499–505. 22354849 10.1002/jbmr.1547 PMC3449092

[em70031-bib-0193] Yamada, M. , R. D. Landes , Y. Mimori , Y. Nagano , and H. Sasaki . 2016. “Radiation Effects on Cognitive Function Among Atomic Bomb Survivors Exposed at or After Adolescence.” American Journal of Medicine 129, no. 6: 586–591. 26477949 10.1016/j.amjmed.2015.09.002

[em70031-bib-0194] Yentrapalli, R. , O. Azimzadeh , A. Sriharshan , et al. 2013. “The PI3K/Akt/mTOR Pathway Is Implicated in the Premature Senescence of Primary Human Endothelial Cells Exposed to Chronic Radiation.” PLoS One 8, no. 8: e70024. 23936371 10.1371/journal.pone.0070024 PMC3731291

[em70031-bib-0195] Yirmiya, R. , and I. Goshen . 2011. “Immune Modulation of Learning, Memory, Neural Plasticity and Neurogenesis.” Brain, Behavior, and Immunity 25, no. 2: 181–213. 20970492 10.1016/j.bbi.2010.10.015

[em70031-bib-0196] Yoshida, K. , H. Oida , T. Kobayashi , et al. 2002. “Stimulation of Bone Formation and Prevention of Bone Loss by Prostaglandin E EP4 Receptor Activation.” Proceedings of the National Academy of Sciences of The United States of America 99, no. 7: 4580–4585. 11917107 10.1073/pnas.062053399 PMC123690

[em70031-bib-0197] Zhang, N. , M. Wang , P. Zhang , and T. Huang . 2016. “Classification of Cancers Based on Copy Number Variation Landscapes.” Biochimica et Biophysica Acta 1860, no. 11: 2750–2755. 27266344 10.1016/j.bbagen.2016.06.003

[em70031-bib-0198] Zhu, Y. , E. Nwabuisi‐Heath , S. B. Dumanis , et al. 2012. “APOE Genotype Alters Glial Activation and Loss of Synaptic Markers in Mice.” Glia 60, no. 4: 559–569. 22228589 10.1002/glia.22289 PMC3276698

[em70031-bib-0199] Zielinski, J. M. , P. J. Ashmore , P. R. Band , et al. 2009. “Low Dose Ionizing Radiation Exposure and Cardiovascular Disease Mortality: Cohort Study Based on Canadian National Dose Registry of Radiation Workers.” International Journal of Occupational Medicine and Environmental Health 22, no. 1: 27–33. 19329385 10.2478/v10001-009-0001-z

[em70031-bib-0200] Zieman, S. J. , V. Melenovsky , and D. A. Kass . 2005. “Mechanisms, Pathophysiology, and Therapy of Arterial Stiffness.” Arteriosclerosis, Thrombosis, and Vascular Biology 25, no. 5: 932–943. 15731494 10.1161/01.ATV.0000160548.78317.29

[em70031-bib-0201] Zou, B. , J. P. Schuster , K. Niu , Q. Huang , A. Rühle , and P. E. Huber . 2019. “Radiotherapy‐Induced Heart Disease: A Review of the Literature.” Precision Clinical Medicine 2, no. 4: 270–282. 35693876 10.1093/pcmedi/pbz025 PMC8985808

